# Valorization of Humins by Cyclic Levulinic Acid Production Using Polyoxometalates and Formic Acid

**DOI:** 10.1002/cssc.202401973

**Published:** 2025-01-28

**Authors:** André Wassenberg, Tobias Esser, Maximilian J. Poller, Dorothea Voß, Jakob Albert

**Affiliations:** ^1^ Institute for Technical and Macromolecular Chemistry University of Hamburg 20146 Hamburg Germany

**Keywords:** Environmental catalysis, Levulinic acid, Humins, Nanofiltration, Polyoxometalates, Formic acid

## Abstract

At a time when increasing attention is paid to sustainability in chemistry, levulinic acid (LA) is one of the most important platform chemicals for the goal of overcoming our dependence on fossil raw materials. However, a so far limiting obstacle on the way to efficient LA production from biomass is the formation of undesirable humin byproducts. In this work, a new catalytic route for the effective utilization of these humin byproducts, enabling a cyclic synthesis of LA using formic acid (FA) as organocatalyst is proposed. Selective catalytic oxidation (SCO) of humins using the H_5_PV_2_Mo_10_O_40_ (HPA‐2) polyoxometalate (POM) catalyst produces FA that can be isolated from the aqueous reaction mixture by using nanofiltration membranes accompanied by a complete catalyst recycling (>99 %). After concentration of FA by distillation, the latter can be used as organocatalyst for LA production from sugars, whereby the formed humins can in turn be separated and used as substrates for further FA production via SCO to close the catalytic cycle. By using FA as a green and sustainable acidic organocatalyst, relatively high yields of LA (up to 42 mol %) could be achieved. In the future this can potentially lead to the creation of a closed cycle for an environmentally friendly and efficient production of green LA without undesired humin formation.

## Graphical abstract

In this work, a new catalytic route for the effective utilization of humin byproducts, enabling a cyclic synthesis of levulinic acid (LA) using formic acid (FA) as organocatalyst is proposed. Selective catalytic oxidation (SCO) of humins using the H_5_PV_2_Mo_10_O_40_ (HPA‐2) polyoxometalatecatalyst produces FA that can be isolated from the aqueous reaction mixture that can be used as organocatalyst for LA production from sugars, whereby the formed humins can in turn be separated and used as substrates for further FA production via SCO to close the catalytic cycle.

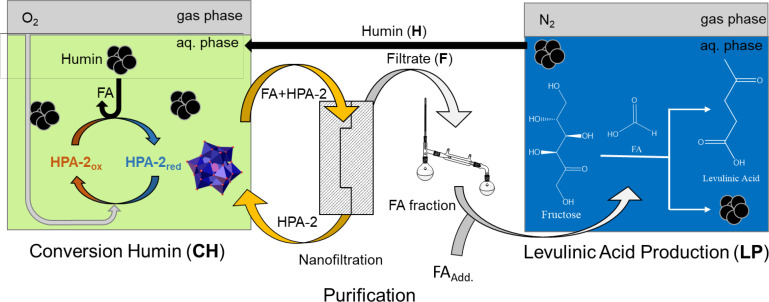

## Introduction

Elimination of environmental pollutants is one of the biggest challenges in the chemical industry of the 21^st^ century. Currently, over 90 % of chemical intermediates are made from crude oil resulting in a need for new sustainable alternatives to replace finite fossil resources in the industry as well as the energy sector.^[1]^ Platform chemicals such as levulinic acid (LA), which can be produced from lignocellulosic biomass, are promising alternatives, as they provide a renewable source of carbon both for energy and environmental applications.^[2]^

LA is an important platform chemical, as it provides highly reactive functional groups granting access to a variety of chemical transformations.^[3]^ The latter can be further processed into commodities, such as γ‐valerolactone,[^4^, ^5^] ethyl levulinate[^6^, ^7^, ^8^] or methyl tetrahydrofuran.^[9]^ Moreover, this includes the production of polymers,^[10]^ pharmaceuticals,[^11^, ^12^] resins,^[13]^ or fuel additives.[^6^, ^14^] While there are different ways to obtain LA such as fermentation of algae or extraction from waste materials,[^15^, ^16^] the most widespread industrially used method is the direct synthesis through the biofine process,^[17]^ where LA is produced together with other chemicals through the acid‐catalyzed conversion of lignocellulosic biomass. Although this process achieves very high LA yields of up to 70 %, it still faces several challenges.^[2]^ One of the primary issues is the use of sulfuric acid as acidic catalyst for the biomass hydrolysis, as it is a strong mineral acid that is difficult to recycle, harmful to the environment, and corrosive to the reaction system.[^2^, ^18^] For this reason, there has been a lot of research into alternative acidic catalysts. For example, acidic resins or zeolites have been investigated as solid acid catalysts as they are easier to separate and less harmful to the reaction systems.[^5^, ^19^, ^20^, ^21^, ^22^] However, despite some progress, none of these processes have been implemented industrially. A more promising strategy would be to use an alternative homogeneous catalyst, that would have to be relatively easy to separate and harmless to the reaction system. Strong organic acids such as formic acid (FA) fit this requirement profile perfectly, but FA‐catalyzed LA synthesis have so far shown far too low yields to be industrially compatible.[^23^, ^24^, ^25^]

Another challenge for the sustainable production of LA is the formation of solid humin residues during the reaction process. Humins are sticky, water‐insoluble by‐products that form during the acidic conversion of cellulose and its derivatives (e. g. glucose, fructose, 5‐hydroxymethylfurfural (5‐HMF)) at temperatures above 140 °C.[^26^, ^27^, ^28^, ^29^] They are dark‐colored spherical particles that are insoluble in conventional solvents,^[30]^ causing problems during industrial processes, as the formation of humins tends to clog up reactors and catalysts.[^22^, ^31^, ^32^] It is assumed that the process of humin formation begins with 5‐HMF, an intermediate that is mainly formed during the acidic conversion of hexoses on the way to LA, and its hydrolyzation product 2,5‐dioxo‐6‐hydroxyhexanal. These further react through esterification, etherification, acetalization, and aldol condensation so that the polymeric humins are formed, whereby other substances such as LA are also incorporated into the humins through polymerization or absorption.[^26^, ^32^, ^33^, ^34^] However, the exact process of humin formation and the exact structure of humins remain unclear to date. What is certain, however, is that the formation of humins is accompanied by a significant loss of carbon, leading to a low atom efficiency.[^33^, ^35^] For this reason, several studies have been carried out to avoid humin formation, for example through the usage of organic solvents.[^32^, ^36^, ^37^, ^38^, ^39^] Although humin formation could be successfully reduced, it has not yet been possible to completely avoid it. Therefore, efforts have been carried out to make sensible use of the humins produced, in order to reduce waste of material, thus increasing the resource efficiency. Humins have been used, for example, for the production of synthesis gas,[^40^, ^41^] as a biofuel additive[^17^, ^42^] or for the production of porous materials.^[43]^ One of the most promising strategies, however, is the conversion of humins into value added chemicals, as the other methods mainly do not attempt to make meaningful use of the more complex organic structures in the humins. The best result would be the recovery of platform chemicals such as 5‐HMF or LA from humins, although this is made difficult by the complex and largely unknown structure of the solids. However, visible progress has already been made in the catalytic depolymerization of the humin structure. Maerten et al.^[44]^ were able to achieve excellent results in 2017 by oxidatively converting humins into FA and acetic acid. FA is an interesting product chemical as, in addition to its synthetic uses, it can also serve as a hydrogen (H_2_) storage medium, with H_2_ being considered as a promising renewable energy source that could replace fossil fuels.^[45]^ Maerten et al. based their work on the commercialized OxFA process,^[46]^ in which polyoxometalate (POM) catalysts are used to produce FA from biomass residues. POMs are inorganic metal oxide clusters whose properties can be adapted to the specific reaction and can catalyze both acid/base and redox reactions.^[47]^ Due to their versatility, they can be used in various catalytic reactions such as the redox reactions in lithium‐sulfur batteries[^48^, ^49^] or the photo‐catalytic oxidation of toluene.^[50]^ POMs are also often used in other green catalytic reactions such as the synthesis of heterocycles^[51]^ or the conversion of biomass into value added products.[^47^, ^52^, ^53^] Out of the different types of POMs, the vanadium containing HPAs have proven to be the most suitable for the conversion of humins, which was shown in a study by Esser et al.^[54]^ During this study the authors determined that the active vanadium species of the HPA‐type POM catalyzes the breakdown of oxygen‐functionalized bonds such as ether and ester groups particularly well. Esser et al. also further refined the selective catalytic oxidation (SCO) of humins introduced by Maerten et al. in multiple studies,[^54^, ^55^, ^56^] resulting in higher carboxylic acid yields and improved atom efficiency.

**Figure 1:**
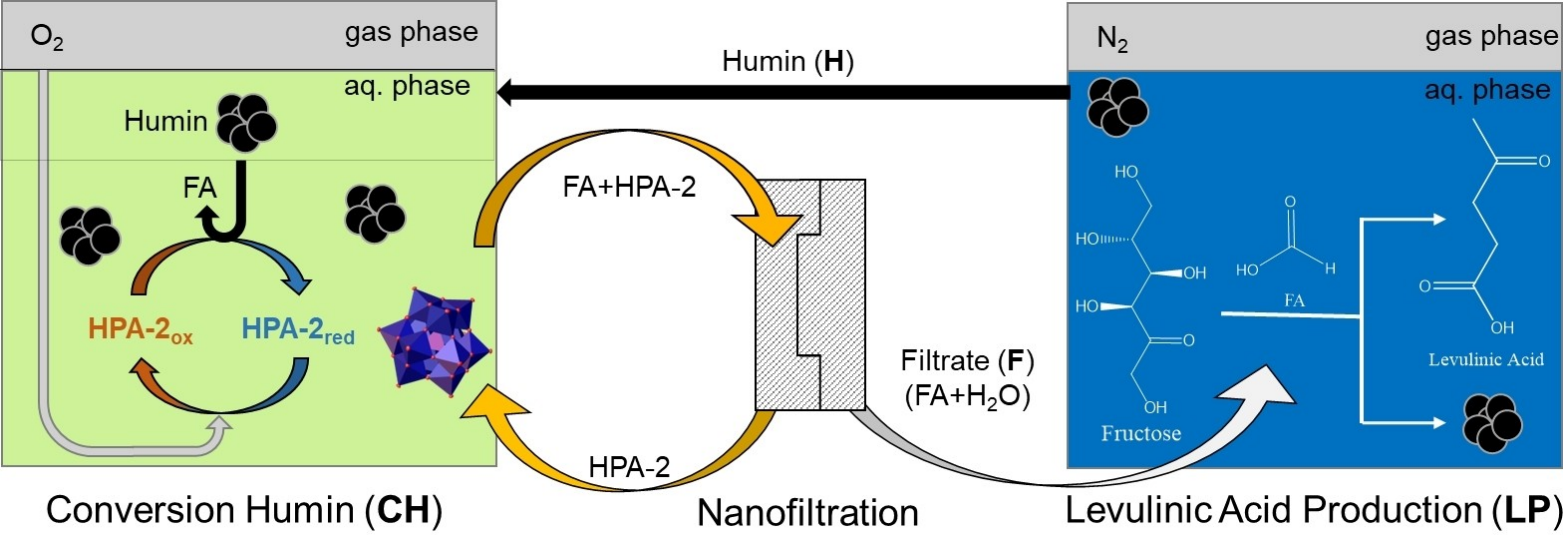
Cyclic LA production from humins for the catalytic elimination of environmental pollutants.

Herein, an innovative cyclic LA production route from humins is proposed using the H_5_PV_2_Mo_10_O_40_ (HPA‐2) POM catalyst for the SCO of humins to FA (**CH**),^[56]^ followed by an efficient downstream separation of FA using nanofiltration,^[57]^ coupled with FA‐catalyzed LA production (**LP**)^[39]^ and recycling of the humin waste (**H**) back into step 1 (**CH**), which would result in a more efficient use of resources (Figure 1).

## Materials and Methods

### Chemicals

Without any additional purification, most of the synthesis‐related compounds were obtained through commercial purchases. Merck Millipore provided the D(+)‐xylose (99 %), D(+)‐glucose (99 %), and D(−)‐fructose (99 %, for biochemistry). Formic acid (>98 %) and sucrose (99 %) were obtained from Alfa Aesar. The source of D(+)‐Cellobiose (>98 %) was Carl Roth. Methanol (99.8 %) was purchased from VWR Chemicals. The HPA‐2 catalyst was synthesized according to a literature‐known synthesis procedure.^[58]^

### Catalyst Characterization

Inductively coupled plasma‐optical emission spectroscopy (ICP‐OES) was used to determine the stoichiometry of the HPA‐2 catalyst using a *Fa. Spectro Arcos (Ametek) ICP‐OES spectrometer*. A stoichiometry of 1.12 P/1.96 V/10 Mo was determined. The integrity of the Keggin‐structure was verified using attenuated total reflection Fourier‐transform infrared spectroscopy (ATR‐FTIR) using a *QATRTM‐S single‐reflection ATR* from *SHIMADZU* (Figure S13), as well as using Nuclear Magnetic Resonance (NMR) ^51^V NMR and ^31^P NMR spectroscopy with a *BRUKER AVANCE II 600 MHz spectrometer* (Figures S14–15). To measure the reaction samples, 0.5 mL of the filtered reaction solutions were combined with 0.1 mL of D_2_O. The used HPA‐2 catalyst was tested for its structural integrity by FT‐IR (Figure S16) as well as its reactive stability by Esser et al. during their previous work using ^51^V‐NMR spectroscopy (Figure S17).^[56]^

### Experimental Setup & Procedure for SCO of Humins (CH)

The SCO experiments were carried out in a 600 mL Hastelloy C276 autoclave with a glas inlet and a gas entrainment stirrer. A method according to Esser et al.^[56]^ was used for the SCO of the produced humins. For this purpose, 1.5 g of the humin was dispersed in 150 mL of a 95 % water/5 % methanol solution and oxidized using 1 mmol of HPA‐2 as a catalyst and 30 bar of pure oxygen as oxidant for 30 h at 1000 rpm.

### Experimental Setup & General Method for Nanofiltration Experiments

The membrane system used was previously described elsewhere.^[57]^ The core of the system is a membrane cell and a HPLC pump from *Bischoff Analysentechnik u. ‐geräte GmbH*, which enables flow rates of up to 40 mL min^−1^ at a pressure of up to 100 bar and is therefore responsible for the pressure build‐up. A special feature of the membrane system is the membrane cell with an active membrane area of 33 cm^2^, which has a stirrer integrated into the cell. The stirrer allows high flow across the membrane and high turbulence regardless of the pump flow rate enabling high concentration factors even with small membrane areas. This reduces phenomena such as concentration polarization. The special design of the membrane cell was developed and patented by PS *Prozesstechnik GmbH*, where the membrane cell is also commercially available.^[59]^ More details on the development, design and optimization of the membrane system for POM separation after SCO of humins can be found in literature.[^56^, ^57^]

The XN45 membrane used is commercially available from *Mann+Hummel*. Before use, the membrane cutout was pre‐wetted inside the membrane cell. For this purpose, the installed membrane was rinsed with water for 5 minutes at a flow rate of 15 mL min^−1^ and a stirrer speed of 1100 rpm. Subsequently, the membrane was rinsed for additional 115 minutes at a pressure of 30 bar. After storage in the flooded cell for 24 hours without pressure, the membrane could be used, as sufficient wetting and swelling could be assumed.^[57]^

In a typical separation, the membrane system was rinsed with the product solution at a flow rate of 15 mL min^−1^ and a stirring speed of 1100 rpm without applying transmembrane pressure for 5 minutes recycling all streams back into the feed storage. Afterwards, the system was rinsed at a pressure of 30 bar for 15 minutes. Subsequently, the purified permeate containing FA was withdrawn into an individual vessel and the catalyst‐rich retentate was recycled back into the feed storage. At the beginning of this process, a sample of the feed solution was taken, and the time measurement was started. The retentate was circulated until half of the original feed solution was drawn off as catalyst‐poor permeate. Finally, the membrane system was cleaned by rinsing with water and air. The sample of the feed solution, retentate and permeate was analyzed using ICP‐OES, NMR, and HPLC. Based on the analyses, the rejection R of a respective component i could be calculated according to Equation (1). The rejection results from one minus the ratio of the measured mass concentration ß_Per(Com i_) of a component i in the permeate to its original mass concentration ß_Feed(Com i)_ in the feed.

(1) $$R_{Com\;i}=1-\frac{\beta_{Per}\left(Com\;i\right)}{\beta_{Feed}\left(Com\;i\right)}$$

The permeate flux J is defined as the mass of permeate m_Per_ withdrawn over the used membrane area A_Mem_ during the experimental period t, as shown in Equation 2.

(2) $$J=\frac{m_{Per}}{A_{Mem}\cdot t}\;\left[kg\cdot h^{-1}\cdot m^{-2}\right]$$

### Experimental Setup & Procedure for Levulinic Acid Production (LP)

For the conversion of several sugars to LA, a 10‐fold screening plant consisting of ten 20 mL autoclaves made of Hastelloy C276 with PTFE gaskets in batch mode were used. Valves, fittings, and pipes were manufactured of stainless steel 1.4571. The plant was equipped with a heating plate, allowing reaction temperatures up to 200 °C and a magnetic stirrer. All reactors were connected to an oxygen supply line. The reaction times provided did not include the approximate 15 minute heating period.

The conditions used during the reactions in the autoclave were modelled after the ideal reaction parameters were determined in a previous study:^[39]^ First, a stock solution of FA dissolved in water was prepared and set to a pH of 1. Then, 7.5 mL of the stock solution were pipetted into a glass liner containing the respective sugar to achieve a 0.1 mol L^−1^ solution. Afterwards, the glass liners were inserted into the reactors and pressurized with nitrogen to prevent the evaporation of the solvent during the reaction. The reactors were heated up to a temperature of 180 °C under a pressure of 40 bar for 1 hour. After reaction, the solution was filtered, and the liquid phase analysed through High Performance Liquid Chromatography (HPLC) and NMR spectroscopy.

### Analysis of Substrates and Reaction Products

The Elemental analyses of the produced humins were performed using a *EURO VECTOR analyzer model EA‐3000*.

A *SHIMADZU HPLC system* equipped with an Aminex HPX‐87H 300 mm×7.8 mm BIORAD column and a refractive index detector from SHIMADZU was used for the quantitative analysis of the aqueous phase (determination of soluble reaction products such as LA, FA and acetic acid). The eluent used was an aqueous sulfuric acid solution with a concentration of 5 mmol L^−1^. The samples were subjected to analysis at 45 °C, 49 bar pressure, and a flow rate of 0.5 mL min^−1^.

The CO_2_ and CO produced during the conversion of humin in the gas phase were measured with a TCD detector of a *VARIAN 450‐GC* on a 2 m×0.75 mm Shin‐Carbon‐column.

For the NMR samples used for quantification of the results of the humin conversion, 0.6 mL of the filtered reaction solution and 0.1 mL of a 10 wt % tert‐butanol/D_2_O solution were used.

## Results and Discussion

In order to establish a cyclic LA production route from humins, the first step is the SCO of humins to FA using the HPA‐2 POM catalyst.^[56]^ HPA‐2 was used as the catalyst of choice, as it was found to be the best catalyst for oxidatively converting humins into FA among the catalysts tested during previous studies.[^55^, ^56^] Other HPA catalysts resulted in poorer conversion rates of the humins or over‐oxidation of the humins into CO_2_. The application of an optimized reaction procedure using methanol as selectivity enhancer efficiently supresses the over‐oxidation of the carbon skeleton to CO_2_ and allows for a higher FA yield as well as selectivity.^[55]^

### SCO of Humins for FA Production Using HPA‐2 as a Catalyst (CH)

**Figure 2:**
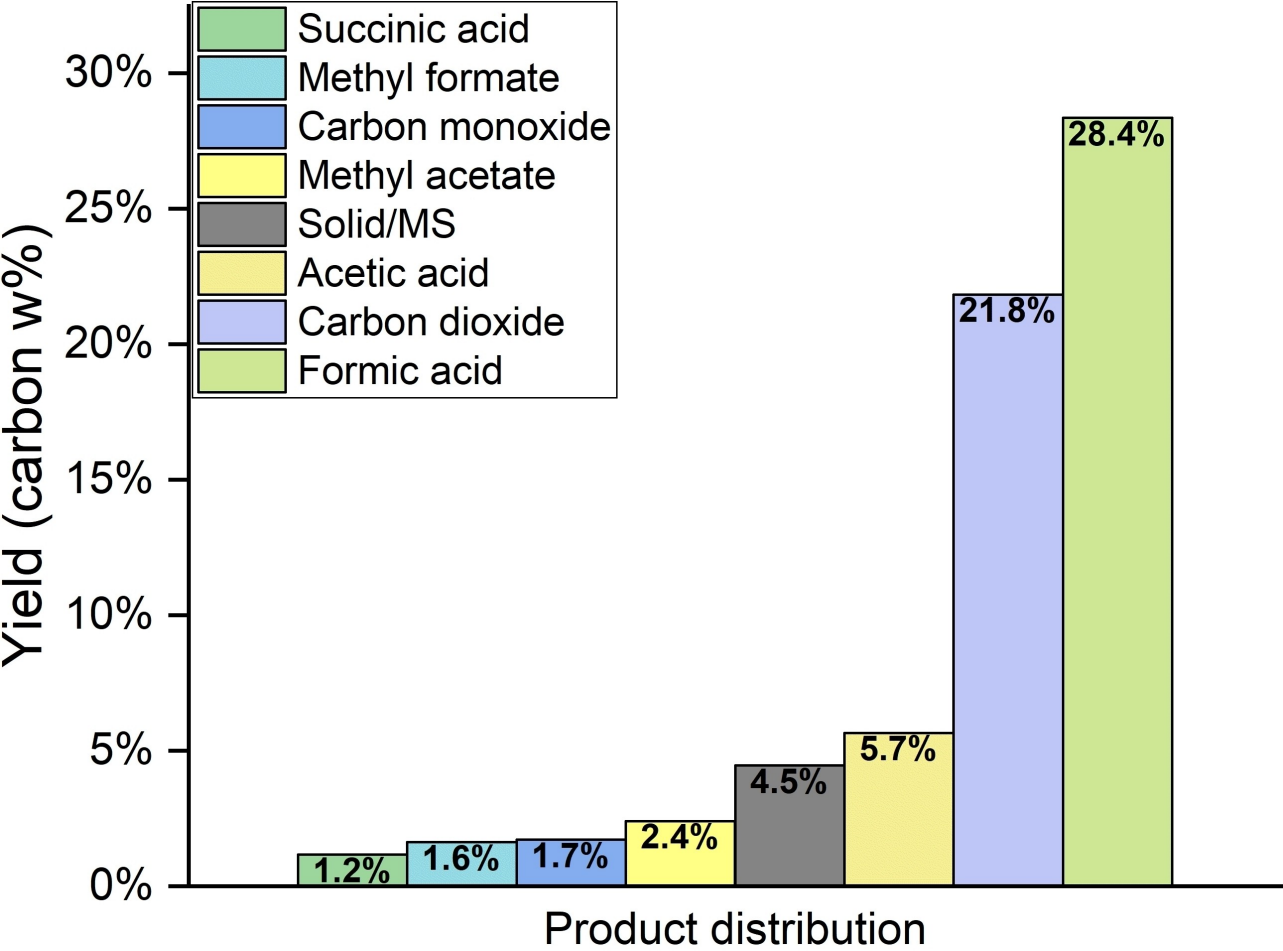
Results for the SCO of humin (90 °C, 30 h, 1 wt % humin in 95 : 5 H_2_O/MeOH solution, p=30 bar O_2_, 2.5 wt % HPA‐2, carbon yields determined by ^1^H‐NMR (Table S5), GC‐MS and using equation S2).

First of all, a significant amount of humin was produced using the procedure described in the supporting information (Table S1, Figures S1 & S2). The as‐synthesized humin was converted by SCO using the HPA‐2 catalyst in the 600 mL autoclave (Figure 2). In contrast to previous research,^[60]^ SCO was not carried out in a pure aqueous solution, but applying an optimized reaction procedure shown by Esser at al.^[56]^ using 5 vol % methanol as an additive to efficiently supress the formation of carbon dioxide.

During the reaction, a conversion of 95.5 % of the solid humin could be achieved, with 4.5 wt % of the humin mass remaining as solid residue. The main product was the desired FA with a yield of 28.4 wt % of the initial carbon. In addition, acetic acid (5.7 wt %), methyl acetate (2.4 wt %), methyl formate (1.6 wt %), and succinic acid (1.2 wt %) were detected in the liquid phase via ^1^H‐NMR (Figure S3). The methyl esters were presumably produced via esterification of the carboxylic acids formed during SCO with the additive methanol. In the gas phase, CO_2_ (21.8 wt %) and CO (1.7 wt %) were found. The total carbon content of the liquid phase was 38.4 wt % while that of the gas phase was 23.5 wt %, respectively, which is a noticeable improvement compared to previous studies.[^44^, ^60^] As no further compounds were detected in the NMR spectra, it is assumed that a large number of smaller carbon compounds were formed that were present in too low amounts to be detectable or were overshadowed by other larger signals, such as the water peak, as it was the case with methanediol and methoxymethanol.

The remaining solid humin was also structurally changed. Elemental analysis and infrared spectra indicate, analogous to previous research, primarily a breakdown of the ether and ester bonds inside and outside the furan rings (Figure S4 and Table S2).^[60]^

### Nanofiltration for HPA‐2 Recycling and FA Isolation Via Distillation

**Figure 3:**
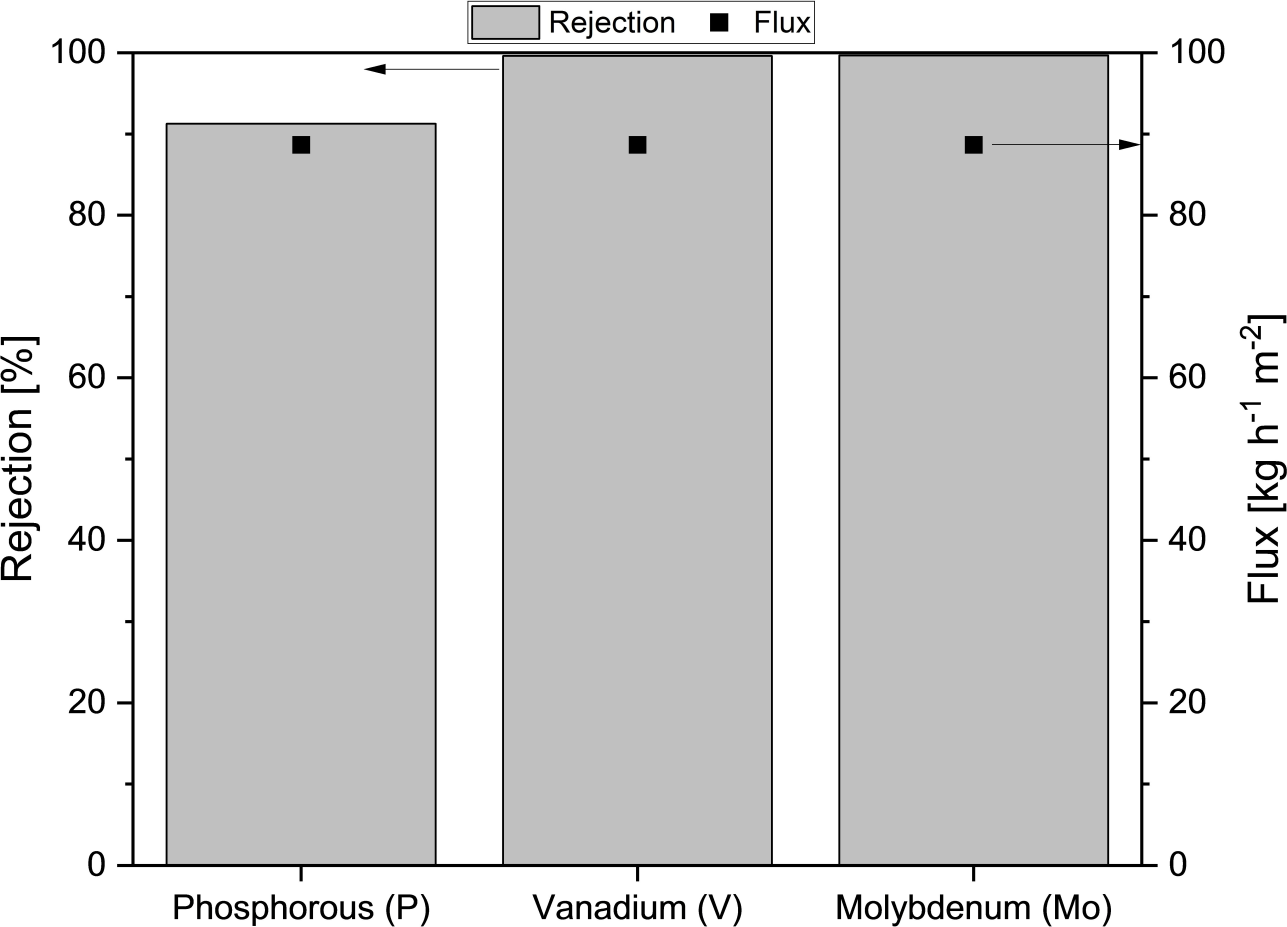
Rejection for catalyst components P, V and Mo in nanofiltration experiments for the product solution of the SCO of humins using HPA‐2 in aqueous‐methanolic solution (95 : 5). Experimental conditions: pre‐wetted membranes, ambient temperature, 30 bar transmembrane pressure, 15 mL min^−1^ flow rate, 1100 rpm stirring speed.

A nanofiltration system, which has already been described in detail in previous studies[^56^, ^57^] was used to separate the HPA‐2 catalyst from the aqueous reaction products. Therefore, the selected membrane must have a different resistance for the components to be separated. Ideally, one component cannot and the other can almost unhindered penetrate the membrane. The resistance of the membrane for a component is described by the rejection for a respective component, whereby a rejection of 100 % means that the membrane is completely impermeable to the respective component. Since the HPA‐2 catalyst should be recycled in the oxidation process, it is desirable that it is completely rejected by the membrane and enriched in the retentate. The acid‐stable nanofiltration membrane XN45 proved to be very promising in previous studies[^56^, ^57^] due to its high selectivity with rejections for the catalyst components of over 99 % as well as low rejection of FA of close to 0 % and thus enables efficient separation of HPA‐2 and product. A detailed description of the experimental methodology for nanofiltration can be found in the corresponding section of the experimental part. Figure 3 shows the results for the rejections of the catalyst and therefore the membrane′s ability to separate the catalyst. Since the HPA‐2 dissociates in the aqueous medium, the catalyst components phosphorous (P), vanadium (V) and molybdenum (Mo) were analyzed individually giving the rejection for each component.

As shown in Figure 3, the XN45 membrane in the nanofiltration system enabled high rejections for V and Mo of over 99 % in the present separation and therefore enables the enrichment of the catalyst in the retentate as well as the production of an almost catalyst free filtrate. The comparatively lower rejection for phosphorous is due to the excess phosphate incurred during the synthesis of the catalyst.^[61]^

Figure S5 shows the changes in composition for the organic compounds. In addition to its high selectivity, the XN45 membrane is also characterized by a high flux of almost 90 kg h^−1^ m^−2^, enabling a fast and efficient downstream processing. By using nanofiltration, the initial catalyst concentration in the product solution could be significantly decreased, resulting in a purified product solution. More specifically, the initial concentration was decreased from over 25 mmol L^−1^to 0.1 mmol L^−1^ for V and from over 127 mmol L^−1^ to 0.4 mmol L^−1^ for Mo, yielding the mostly catalyst free filtrate. Overall, the promising results of the previous studies were reproducible.^[56]^ Furthermore, in these studies it was also successfully demonstrated that the catalyst is recyclable using nanofiltration and the selected XN45 membrane.

**Figure 4:**
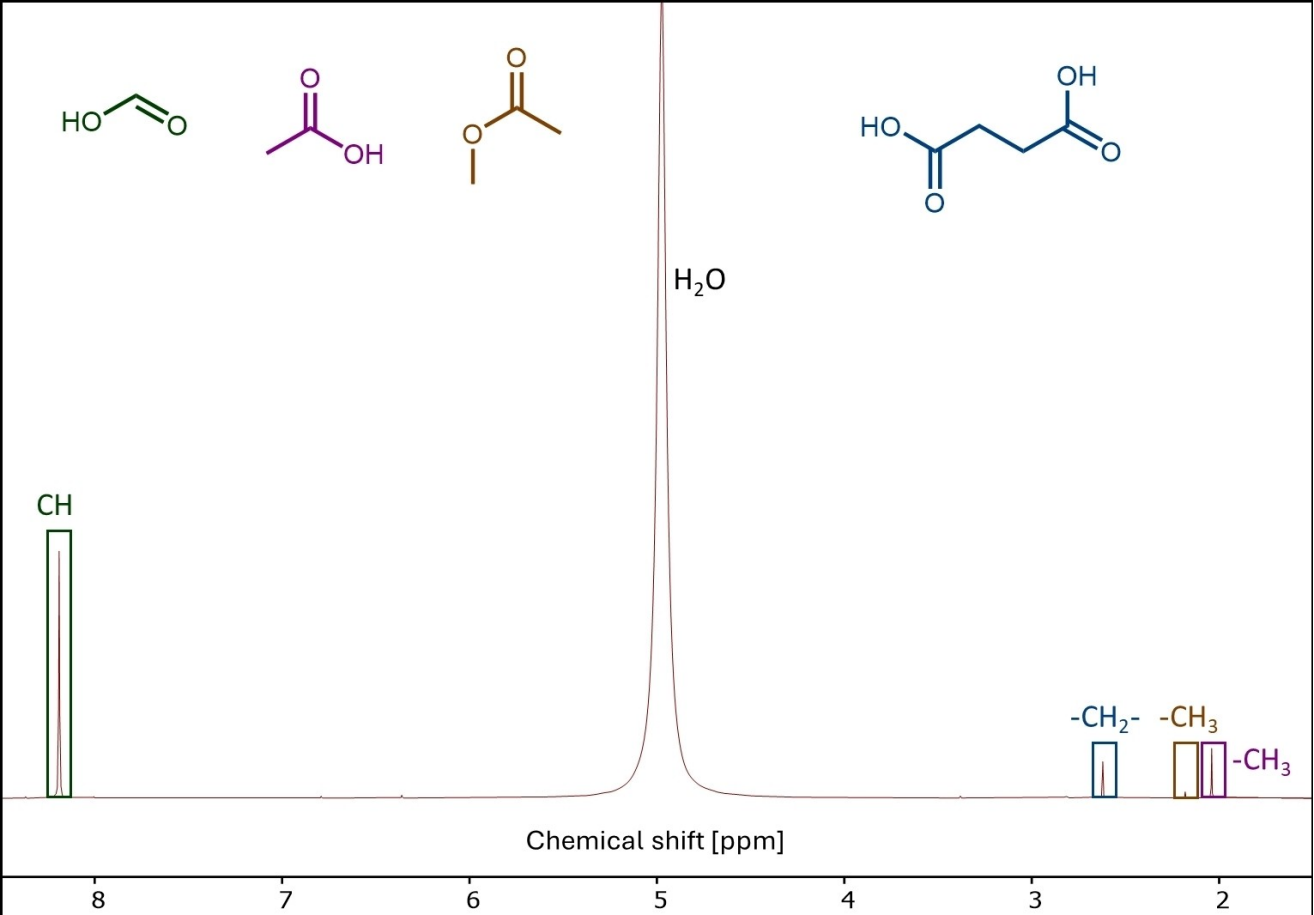
^1^H‐NMR of the FA‐fraction after atmospheric distillation.

After nanofiltration, the filtrate was additionally separated using distillation to achieve a pure FA solution, as the membrane treatment did not remove all impurities from the reaction solution. Additionally, the measured pH value (2.1) of the permeate was deemed too high for the consecutive **LP** reaction for LA synthesis which would have to be carried out under a pH value of almost 1. The reaction solution was therefore distilled over 12 hours at up to 130 °C under atmospheric pressure in order to separate the obtained FA from the rest of the reaction solution and to further concentrate it to the distinguished acidity required for the **LP** reaction. After distillation, the highest concentration of FA could be found in the last fraction, which was left at the bottom of the flask, which is referred to hereafter as FA‐fraction (Figure 4). While it was possible to completely remove most of the volatile compounds such methanol or methylformate, it was not possible to remove all other reaction products from the FA‐fraction (Figures S6 & S7) due to their higher boiling points like succinic acid.

After distillation, the FA‐fraction consisted of 87.00 wt % water, 11.65 wt % FA, 0.73 wt % succinic acid, 0.49 wt % acetic acid and 0.13 wt % methyl acetate. The pH value of the reaction solutions for **LP** was approx. 1 while the pH value of the fraction reached about 1.3.

### Levulinic Acid Production (LP) Using FA as Organocatalyst

**Figure 5:**
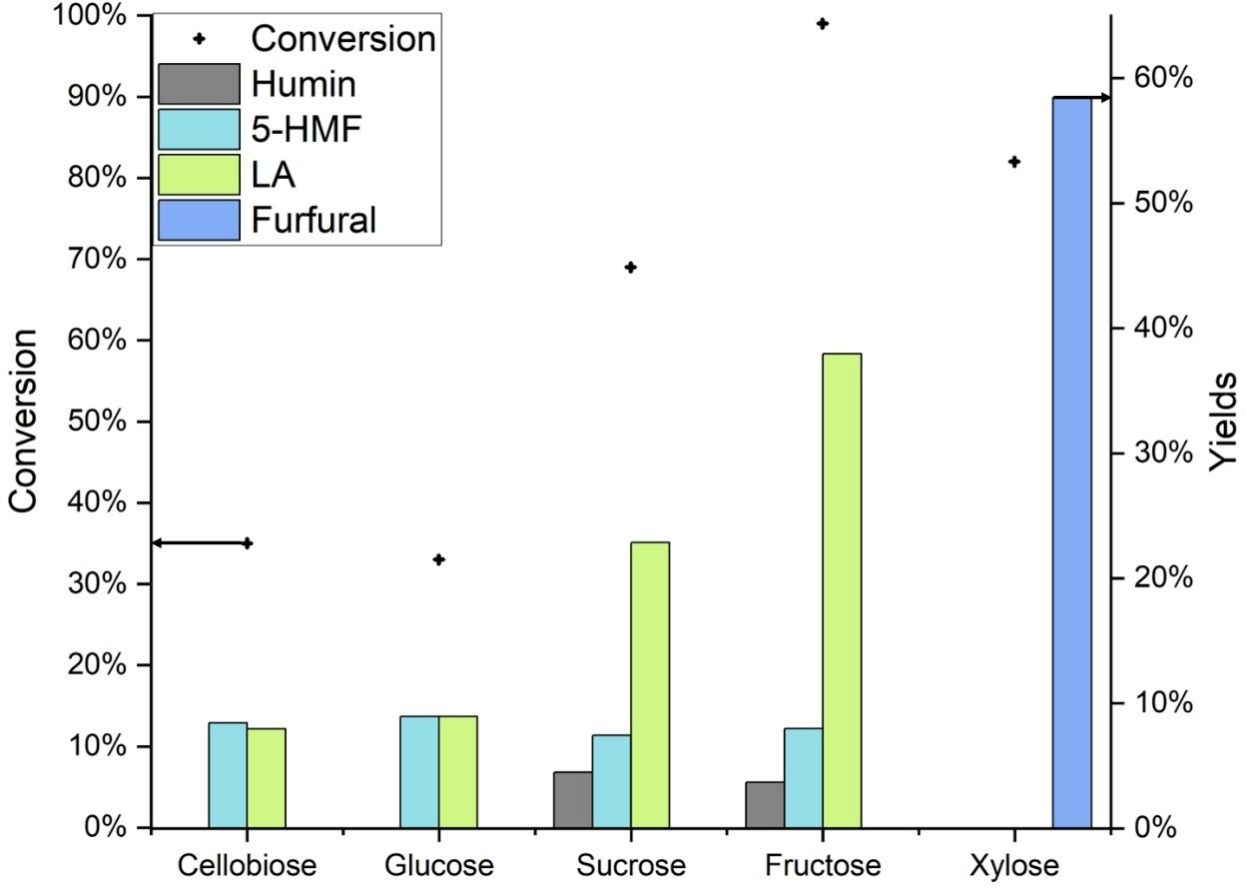
Conversion of various sugars using FA as an organocatalyst. Yields of LA (mol %), 5‐HMF (mol %), furfural (mol %) and humin (wt %). Reaction conditions: 0.1 mol L^−1^ sugar, 7.5 mL H_2_O, pH=1 (FA), 180 °C, 1 hour, p=40 bar N_2_, yields determined by HPLC.

In order to establish a cyclic levulinic acid production (**LPC**) from humins, a suitable carbohydrate source for the initial LA synthesis using FA as an organocatalyst must be found. Similar to previous work,^[39]^ five different sugar derivatives were selected as substrates: Cellobiose and sucrose as hexose‐based disaccharides, their monomers fructose and glucose as well as the pentosic monosaccharide xylose. The results are summarized in Figure 5 and Table S3.

Fructose achieved the highest LA yield of 38 % followed by sucrose with 23 %. Glucose and cellobiose, however, only achieved LA yields of 9 % and 8 %, respectively. This might be due to the fact that glucose requires an additional isomerization step to fructose before it can be hydrolyzed to LA.^[62]^ Contrasting to previous research,^[39]^ significant amounts of 5‐HMF (around 8 % for all hexoses) could also be detected in the reaction solutions, although this can be attributed to the relatively lower acid strength of FA as an acidic catalyst compared to H_4_SiW_12_O_40_, resulting in a lower conversion of 5‐HMF to LA within the short reaction time of 1 hour. Interestingly, only in the conversion of sucrose and fructose a measurable amount of solid humins (around 4 wt %) was formed. When glucose and cellobiose were converted, almost no solid humins were formed. As no further substances were detected in the NMR spectra and the HPLC chromatograms (Figures S8 & S9) for all sugars, it is assumed that, as already postulated by Tsilomelekis et al.,^[32]^ multiple water‐soluble oligomers are formed from the remaining converted sugar, with the relative proportion of oligomers formed being greatest in fructose, the only sugar which was converted completely within the reaction time of 1 hour.

Xylose as the only pentose tested here shows a completely different behavior as it neither produces HMF nor LA or any solid residues. In contrast, only furfural with a yield of 59 % is formed. The formation of furfural from xylose was expected, as xylose is known to form furfural when hydrolyzed under similar conditions.[^63^, ^64^] A formation of LA did not occur as the catalytic hydrogenation of furfural to furfuryl alcohol that is necessary for the formation of LA from furfural was not possible under the conditions applied. Therefore, fructose was chosen as the substrate for initial LA synthesis catalyzed by the organocatalyst FA, as it showed the highest yield of LA.

**Figure 6:**
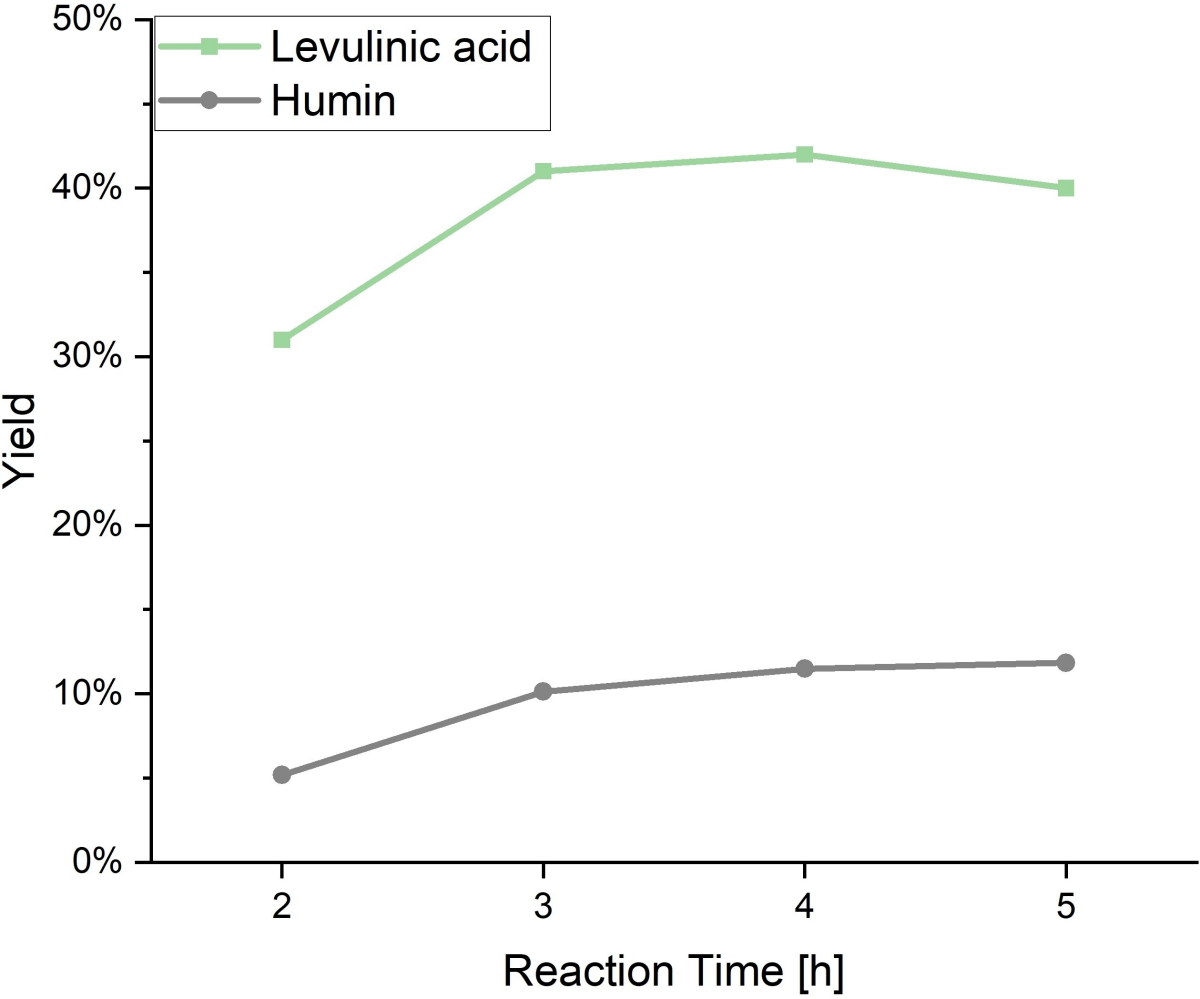
Course of the reaction for the upscaled LA synthesis from fructose in the 600 mL autoclave. LA yields (mol %) and humin yields (wt %) at different reaction times. Reaction conditions: 0.1 mol L^−1^ fructose, 300 mL H_2_O, pH=1 (FA), T=180 °C, p=30 bar N_2_.

After selecting an appropriate substrate, the reaction was scaled up from 20 mL reactors to the larger reactor system (600 mL) in order to test the scalability of the reaction and to produce the necessary amounts of material for the desired cyclic LA production. The synthesis conditions could therefore not be adopted exactly from the previous test, as the larger reactor had a different heating profile. For this reason, an additional series of tests was carried out to adapt the reaction time for the larger scale reactor and to obtain the maximum LA yield. The course of the reaction in the up‐scaled LA synthesis from fructose in the 600 mL autoclave can be seen in Figure 6 and Table S4.

It can be seen that the optimal LA yield is not achieved within 1 hour reaction time, as the LA yield increases up further to reach its maximum with 42 mol % at a reaction time of 4 hours, whereas afterwards it slightly drops down. This drop in LA yield can be traced back to an increasing incorporation of the LA into the humin structure the longer the reaction proceeds. The achieved LA yield at a reaction time of 4 hours is also significantly higher than the highest value achieved during the substrate selection within 1 h reaction time. The fact that larger amounts of 5‐HMF, similar to the previous reaction, were found only in the liquid phase of the reaction at the shortest reaction time (Figure S10), confirms the previous suspicion, which is also further reinforced by an examination of the solid humins produced. Within the first 3 h, the humin yield increases continuously up to 10.1 wt %, whereas from 3 h onwards only a slight increase in yield up to 11.8 wt % could be observed. Elemental analysis (Table S5) and infrared spectroscopy (Figure S11 & Table S6) show strong differences between the humins produced after 2 hours and the other humins after 3 hours onwards, all being very similar to each other, indicating that the solid humins formed undergo several structural modifications during reaction.

With longer reaction times, however, LA could be detected as the sole reaction product in liquid phase confirming the effectiveness of FA as an organocatalyst for selective LA production. Consequently, the ideal reaction time for establishing a cyclic LA production where the organocatalyst FA is directly produced from the humin residue of the original LA synthesis from fructose is considered to be 4 hours, as this reaction resulted in the highest LA yield. Moreover, significant amounts of humin were also produced, allowing for their use as substrate in the SCO, with this reaction hereinafter being referred to as **LP**. In a future industrial process, it would also become relevant to separate the LA from the rest of the solution after the successful synthesis. This is generally achieved through distillation or extraction.^[65]^ Additionally, it could also be advisable to further optimize the FA‐catalyzed LA synthesis in a follow‐up study. The different reaction behaviour of FA compared to the POM catalyst used in the previous study,^[39]^ shows a potential for improvement to increase the FA yield through a further variation of the reaction parameters.

### Cyclic Levulinic Acid Production (LPC) Using a Closed Reaction Cycle

In the next step, reaction **LPC** was carried out, which was intended to emulate a new LA production using the FA obtained from the SCO, thus starting a new cycle. For this purpose, a reaction solution was prepared that corresponded to the composition of the FA‐fraction, as the amount of solution obtained after distillation was not sufficient to carry out the reaction on the same scale as the original LA synthesis **LP**. **LPC** was then carried out according to the parameters of **LP**. In addition, another reaction was carried out with a pure FA/water solution, whereby the FA to water ratio was the same as the ratio of the two substances used in **LPC**. This was intended to serve as a comparison to check what influence the additional substances left in the final fraction had on the reaction and will be referred to as **LPC‐2**. The results compared to the averages of the **LP** reactions can be seen in Table 1.

**Table 1:** Comparison of pH values of reaction solutions and average yields of **LP** with **LPC** and **LPC‐2** pH values and yields (180 °C, H_2_O, 0.1 mol L^−1^ fructose, t=4 h, p=30 bar N_2_).

	LA yield mol %	Humin yield wt %	pH [−]
**LP (avg)**	40.7	11.5	1.0
**LPC**	37.0	11.4	1.3
**LPC‐2**	36.0	12.6	1.3

It can be seen that there are only minor differences between the **LPC** and **LPC‐2**. The LA and humin yields differed slightly, but these differences were still within the range of variance that could already be seen during **LP**. No major differences can be seen in the ^1^H‐NMRs either, apart from the presence of the extra substances in the reaction solution of **LPC** and some additional methanol (Figure S11). The same also applies to the comparison of the spectra of **LP** with **LPC** and **LPC‐2**. The only difference here is the slightly lower LA yield, although this is presumably caused by the lower FA concentration, as the LA formation is known to increase with higher concentrations of the acidic catalyst.^[39]^

The infrared spectra of the humins do not show any discernible differences (Figure S12). It seems like the lower acid concentration and the added substances had no significant impact on the humin formation to significantly alter the humin composition. It can therefore be assumed that it is possible to convert humins in a similar way to SCO and thus making a continued cycle possible.

**Figure 7:**
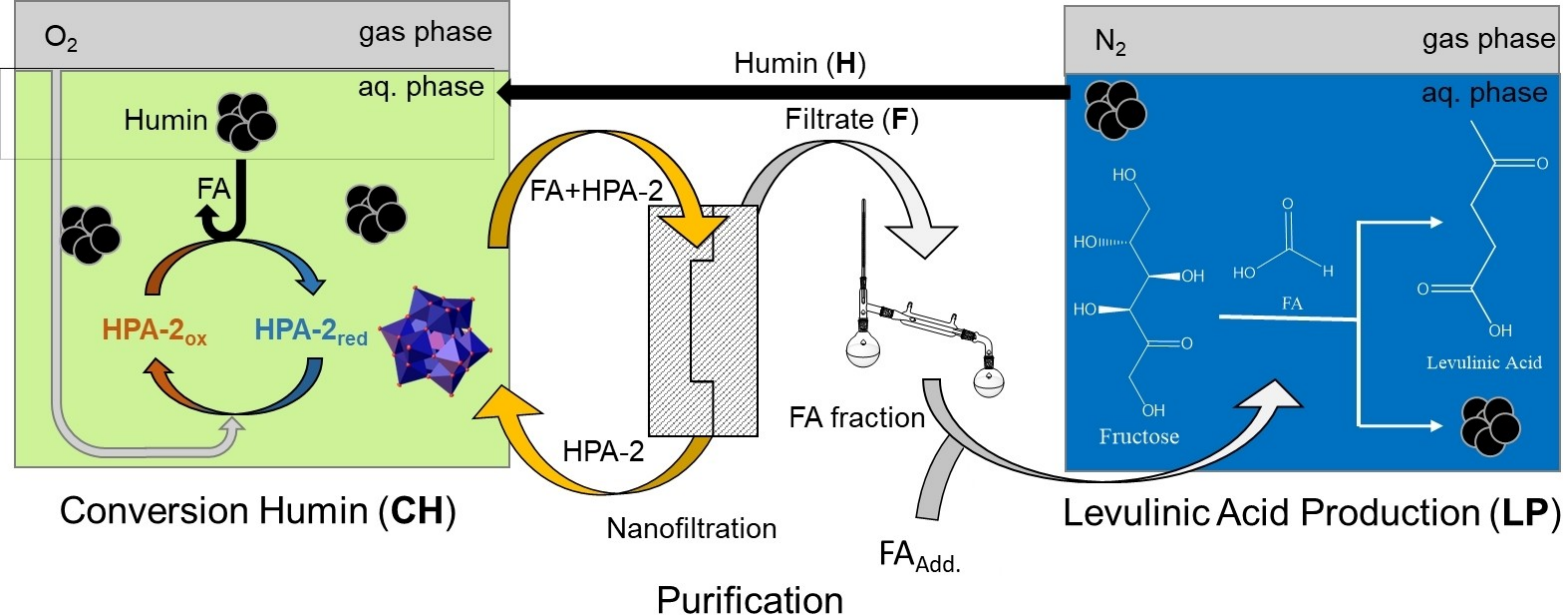
Modified schematics for the cyclic LA production route as performed during this study.

Due to the additional step required during purification, the original planning of the cycle from Figure 1 had to be slightly modified. The reworked cycle is displayed in Figure 7.

The original LA synthesis through **LP** was successful. Yields of an average of 40.7 mol % (1.2 % standard deviation) were achieved in several reactions, with an average humin yield of about 11.5 wt % (0.2 % std. dev.) showing a higher LA and lower humin yield than a comparable experiment from previous research,^[39]^ which underlines the successful optimization of the reaction parameters for LA synthesis. In this case, however, a higher humin yield could be acceptable as long as the LA yield does not suffer, as it would result in more material to be recycled into FA. A higher sugar concentration could possibly be beneficial for future studies. The experiment also had to be carried out several times in order to produce a sufficient amount of humin **H** for further reactions. In the following SCO of humin, FA could be obtained as the main product with a carbon weight content of 28.4 %. This confirmed the previous results of Esser et al.^[56]^ Moreover, the membrane could completely retain the HPA‐2 catalyst and retain its structure (Figure S15). An additional distillation of the permeate had to be carried out in order to increase the FA concentration and to remove additional low‐molecular by‐products. Finally, a cyclic LA production **(LPC)** using a reaction solution whose composition was equivalent to that of the FA‐fraction from distillation was then carried out successful. Although the LA yield of **LPC** was slightly lower with 37 mol %, due to the higher pH value compared to **LP**, it proved the possibility of a continued cycle, as the humins from both reactions showed no discernible differences, despite the additional substances in the reaction solution of **LPC**.

An additional **LPC‐2** with the same pH value but without the low molecular weight impurities in the FA‐fraction did not show any major differences compared to **LPC**. A better distillation would therefore mostly increase the recovery efficiency of the produced FA resulting in a more precise re‐creation of the reaction conditions of **LP**, which should then result in similar results and make the further cyclic process possible. A cyclic synthesis of LA recycling the by‐product into new catalysts is therefore certainly possible, even if some small hindrances have to be eliminated in order to increase the overall efficiency.

## Conclusions

From a selection of five different sugars, fructose was confirmed to be the best substrate for FA‐catalysed LA production. While xylose showed by far the highest conversion, the resulting platform chemical was furfural instead of LA. This combined with a lack of measurable humin residue made xylose unsuitable for the proposed process. Upon subsequent transfer to a larger reactor system, it was proven that the results were scalable to bigger reactors, although the results indicated higher reaction times being beneficial. Further studies into the optimal reaction conditions for the upscaled LA synthesis could also potentially result in even higher LA yields. With the modified reaction parameters multiple reactions were carried out, resulting in a sufficient amount of humin being produced, which was successfully converted by SCO using HPA‐2 to form a larger amount of FA. The subsequent separation from the catalyst via nanofiltration was carried out successfully, although further purification of the permeate by distillation was necessary. In a final step, by using a reaction solution of the same composition as the solution obtained at the end of the distillation, it was proven that a new LA synthesis is possible using the FA‐fraction. It was also confirmed by an additional experiment that the impurities of the FA‐fraction had no noticeable influence on product formation. It could therefore be shown that the formation of a cyclic LA synthesis process using FA as an organocatalyst produced from humin residues from the initial LA synthesis from sugars is chemically possible, even if the process shown here needs to be further optimized. In follow‐up studies, it would also be advisable to carry out a life cycle as well as a technoeconomic analysis of the process in order to examine its economic viability. We believe that these results show a lot of potential and that through improvements in efficiency, for example during the separation, could lead to the production of LA through FA in an environmentally friendly, cyclic system being a potential economical alternative.

## Author Contributions

A. Wassenberg: methodology (main), conceptualization (equal), writing (main); M. J. Poller: methodology (supporting), conceptualization (supporting), supervision, writing – review & editing; T. Esser: methodology (supporting), conceptualization (equal), writing – description of the nanofiltration system and process; D. Voß: project administration (supporting), supervision, writing – review & editing; J. Albert: project administration (main), funding acquisition, resources, writing – review & editing

## Conflict of Interests

The authors declare no conflict of interest.

1

## Supporting information

As a service to our authors and readers, this journal provides supporting information supplied by the authors. Such materials are peer reviewed and may be re‐organized for online delivery, but are not copy‐edited or typeset. Technical support issues arising from supporting information (other than missing files) should be addressed to the authors.

Supporting Information

## Data Availability

The data that support the findings of this study are available from the corresponding author upon reasonable request.
