# Supplementary material for: Valorization of Humins by Cyclic Levulinic Acid Production Using Polyoxometalates and Formic Acid
Source: ChemSusChem. 2025 Jan 28;18(10):e202401973. doi: 10.1002/cssc.202401973 (PMC12094137; doi:10.1002/cssc.202401973)
Supplement: Supplementary file 1 — Supporting Information [file CSSC-18-e202401973-s001.pdf]

ChemSusChem

Supporting Information

Valorization of Humins by Cyclic Levulinic Acid Production Using Polyoxometalates and Formic Acid

André Wassenberg, Tobias Esser, Maximilian J. Poller, Dorothea Voß, and Jakob Albert*

Valorization of Humins by Cyclic Levulinic Acid Production Using Polyoxometalates and Formic Acid

Supporting Information

André Wassenberg, Tobias Esser, Maximilian J. Poller, Dorothea Voß, Jakob Albert[*]

Institute for Technical and Macromolecular Chemistry, University of Hamburg, 20146 Hamburg, Germany

* Correspondence: jakob.albert@uni-hamburg.de

Table of Contents

1. Synthesis of humins for SCO experiments 2
2. SCO of humins (CH) 4
3. Nanofiltration and distillation 6
4. Levulinic acid production (LP) using FA as organocatalyst 8
5. Cyclic Levulinic acid production (LPC) 12
6. Equations 17

1. Synthesis of humins for SCO experiments

Humin synthesis was carried out applying a modified and upscaled version of the optimal reaction conditions for LA synthesis determined in our previous work [1]. The synthesis was conducted in a 600 mL Hastelloy autoclave under a nitrogen atmosphere, with formic acid as acidic catalyst and a reaction time of 4 h. The reaction solution was stirred (300 rpm), the reaction temperature set to 180 °C, and the pressure increased to 30 bar to prevent evaporation of the solvent. The solid humin contained in the reaction solution was separated from the solution through filtration. The solid residue was subsequently rinsed with water and dried.

Table S1: Humin and LA yields of multiple batches for humin synthesis (180 °C, H_2O, 0.1 mol L^{-1} fructose in 150 mL H_2O, pH = 1 (FA), t = 4 h, p = 30 bar N_2).

	LA yield mol%	Humin yield wt%
**LP1**	42.0	11.5
**LP2**	39.0	11.8
**LP3**	41.0	11.4
**Average**	40.7	11.5
**Standard deviation**	1.2	0.2

The yields of humins and LA differ only slightly, which also shows the reproducibility of the reaction. There were also no differences observed in the analysis of the solid and liquid phases (*Figure S1/S2*). For this reason, the three humin batches produced were combined to form humin batch **H** used for the SCO.

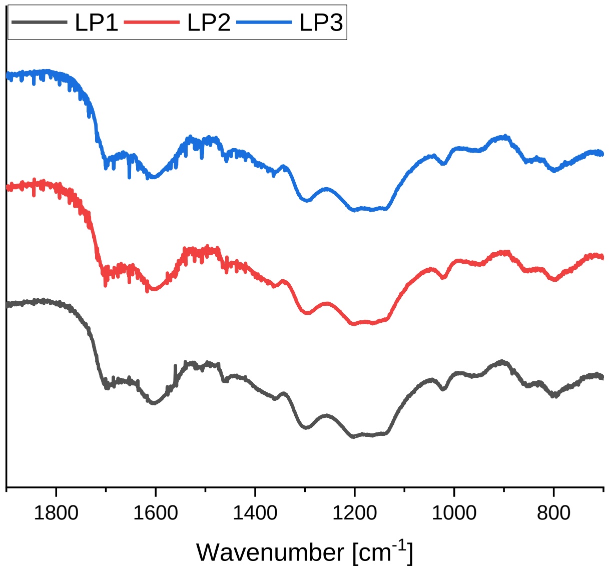

*Figure S1: IR-spectra of fructose humins produced by **LP1**, **LP2** and **LP3** (180°C, 0,1 mol L^{-1} fructose, pH = 1 FA, t = 4h, p=30 bar N_2).*

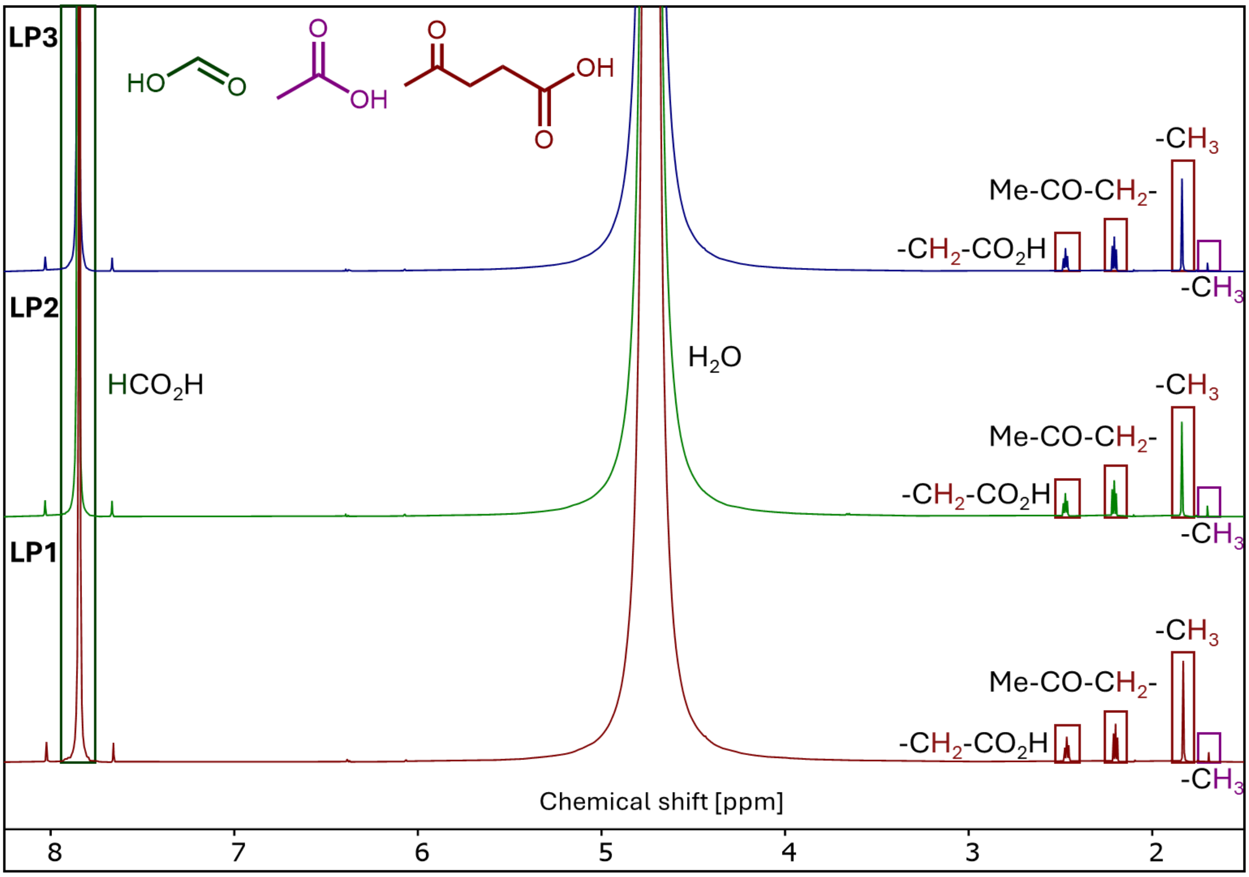

*Figure S2: ^{1}H-NMR (D_2O) spectra of the liquid phase of **LP1**, **LP2** and **LP3** (180°C, 0.1 mol L^{-1} fructose, pH = 1 FA, t = 4h, p=30 bar N_2).*

2. SCO of humins (CH)

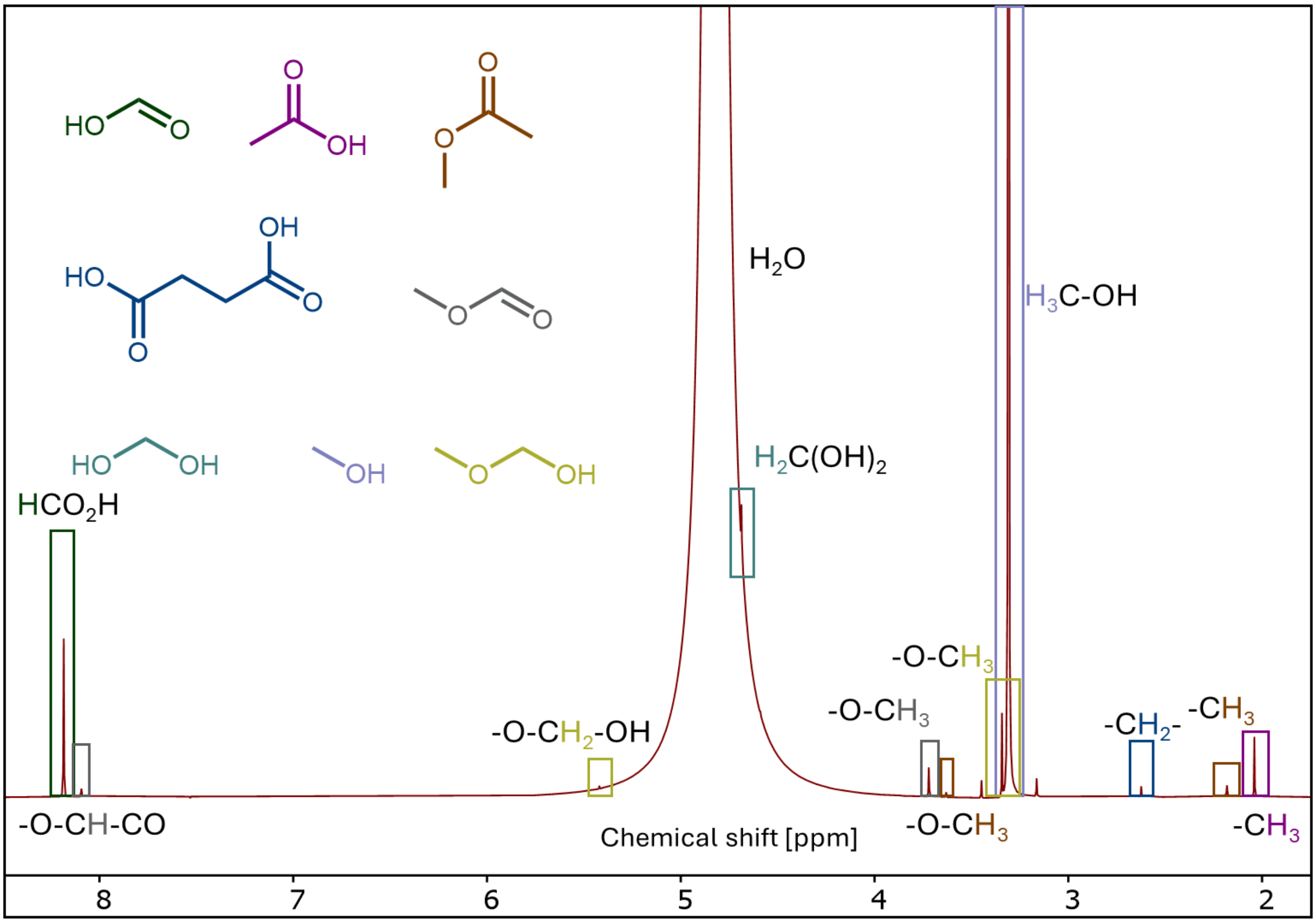

Figure S3: ^{1}H-NMR of the liquid phase of ***CH*** *(90 °C, 30 h, 1 wt% humin in 5% MeOH/H_2O solution, p = 30 bar O_2, 2.5 wt% HPA-2).*

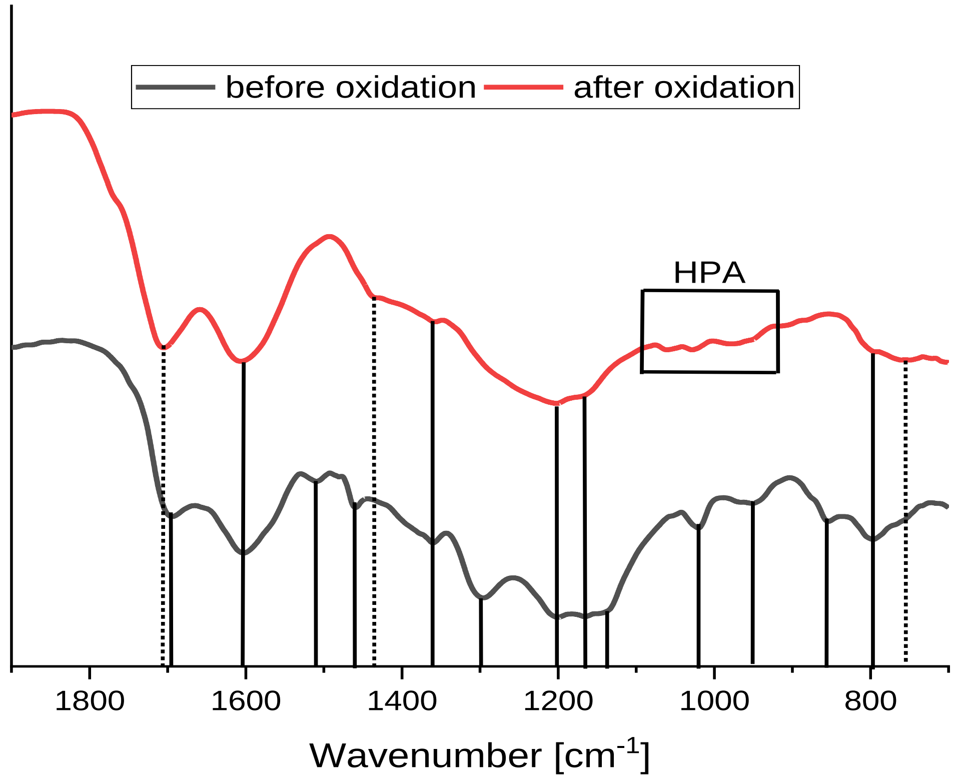

*Figure S4**:** IR-spectra of humine before and after SCO (90°C, 30h, 1 wt% humin in 150 mL 5% MeOH/H_2O solution, p = 30 bar O_2)*

*Table S2: Elemental Analysis of **H** before and after the oxidative conversion (90°C, 30h, 1 wt% Humin in 150 mL 5% MeOH/H_2O solution, p = 30 bar O_2)*

**Elemental Analysis**	**Before**	**After**
**C (w%)**	63.1	53.2
**H (w%)**	4.1	3.9
**O (w%)**	31.2	39.3
**Sum (w%)**	98.4	96.4

3. Nanofiltration and distillation

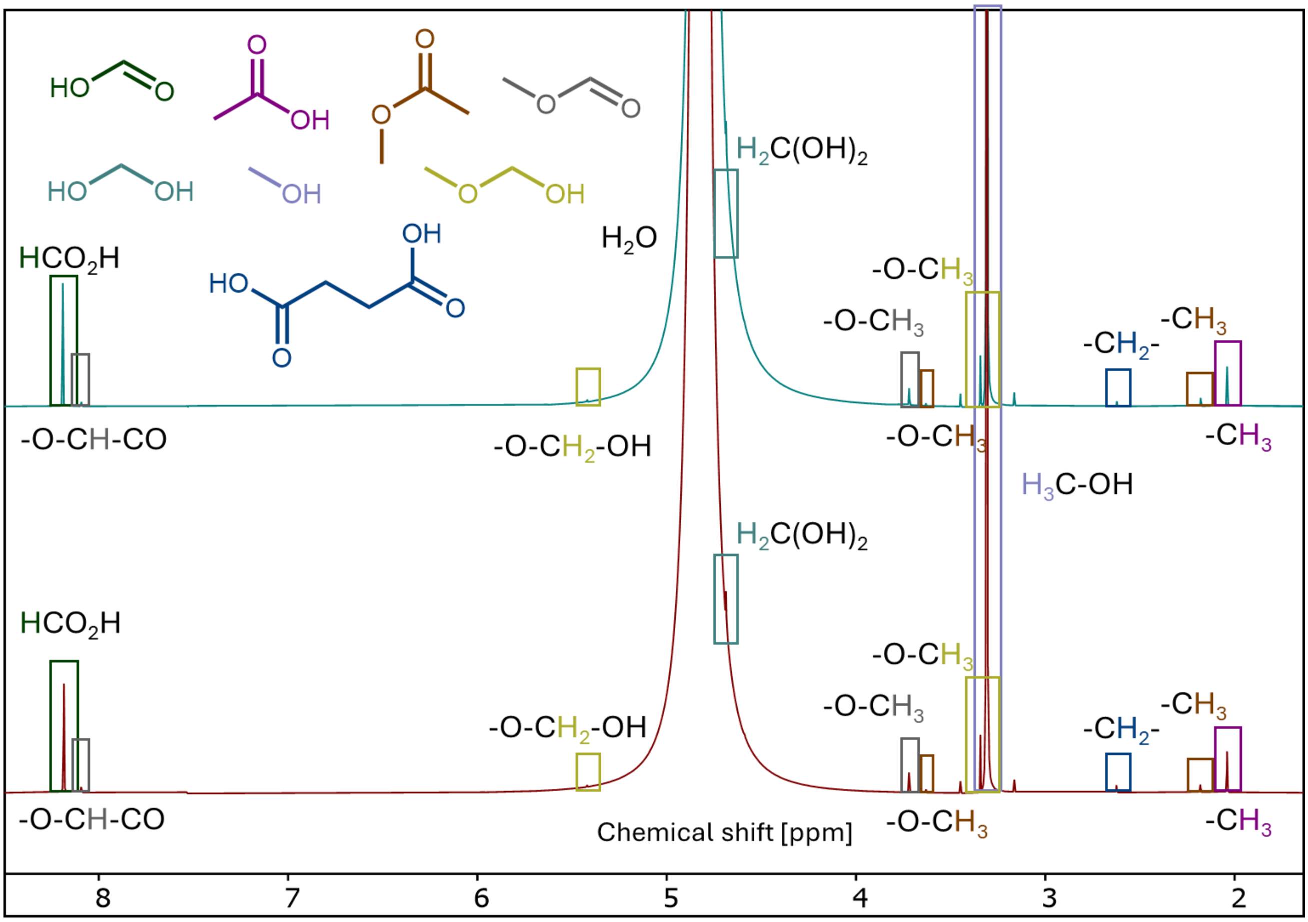

Figure S5: ^{1}H-NMR (D_2O) of the reaction solution of the SCO of humins before (bottom) and after (top) nanofiltration.

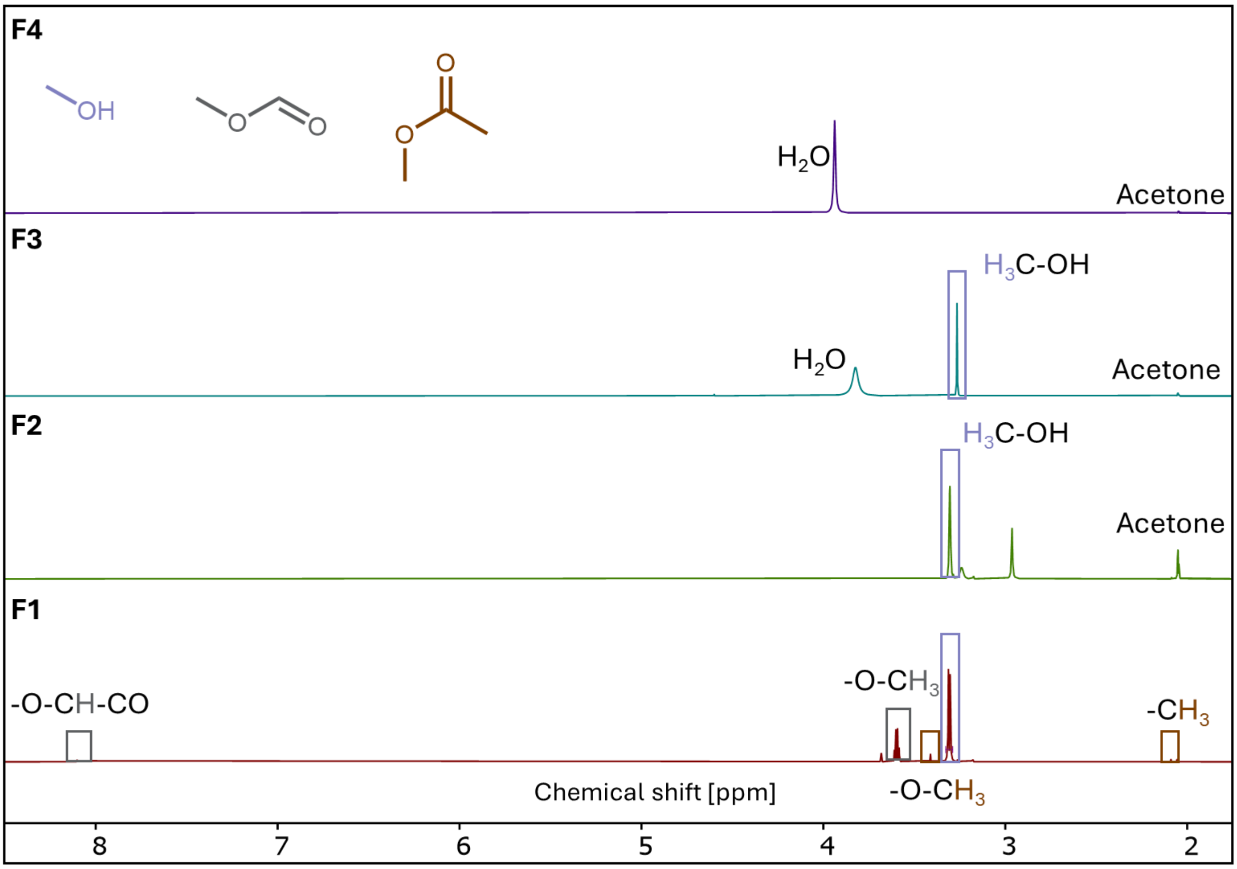

Figure S6: ^{1}H-NMR (Acetone-d_6) spectra of distillation fractions 1-4

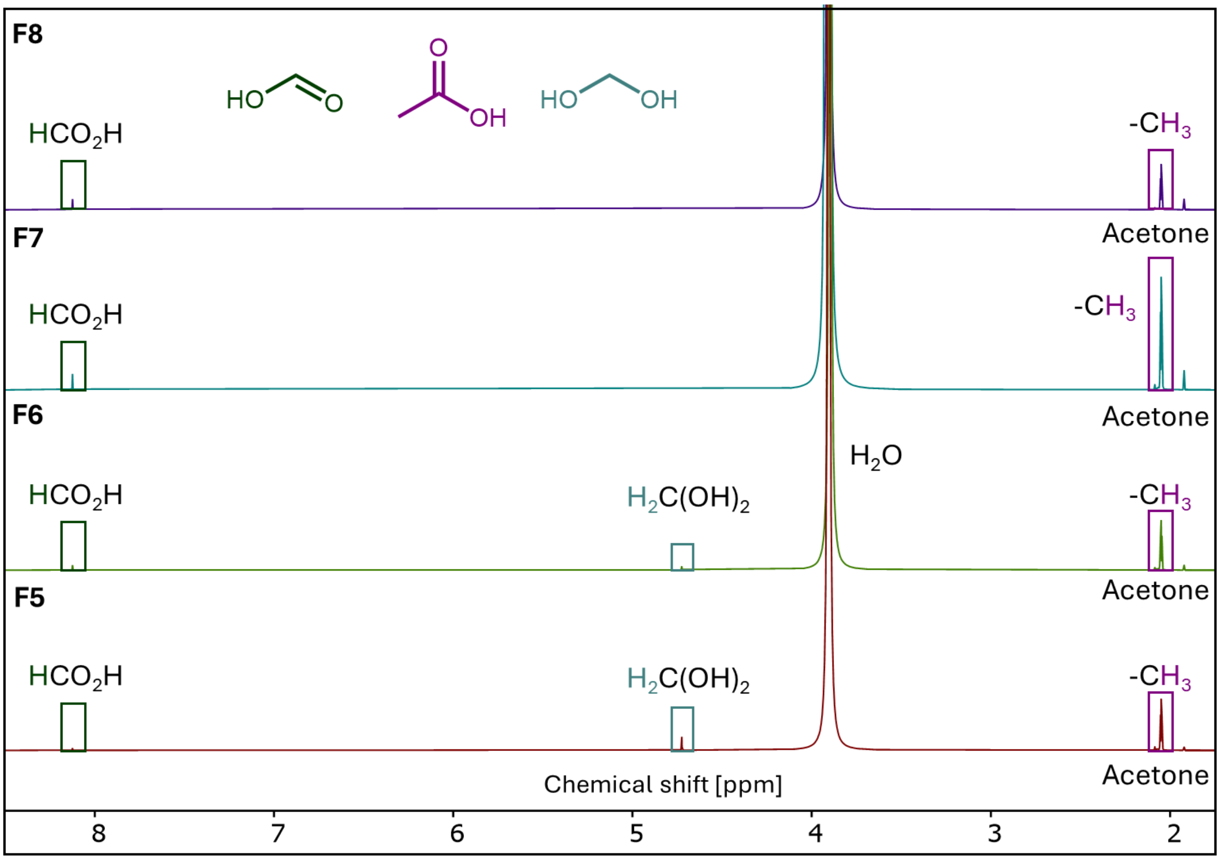

Figure S7: ^{1}H-NMR (Acetone-d_6) of distillation fractions 5-8

4. Levulinic acid production (LP) using FA as organocatalyst

Table S3: Product yields of sugar variation reactions (0.1 mol L^{-1} sugar, 7.5 mL H_2O, pH=1 (FA), 180°C, 1 hour, p = 40 bar N_2).

	Humin (w%)	HMF (mol%)	LA (mol%)	Furfural (mol%)
**Fructose**	3.70%	7.99%	37.95%	0.00%
**Glucose**	0.00%	8.96%	8.96%	0.00%
**Xylose**	0.00%	0.00%	0.00%	58.47%
**Sucrose**	4.48%	7.46%	22.87%	0.00%
**Cellobiose**	0.00%	8.45%	7.95%	0.00%

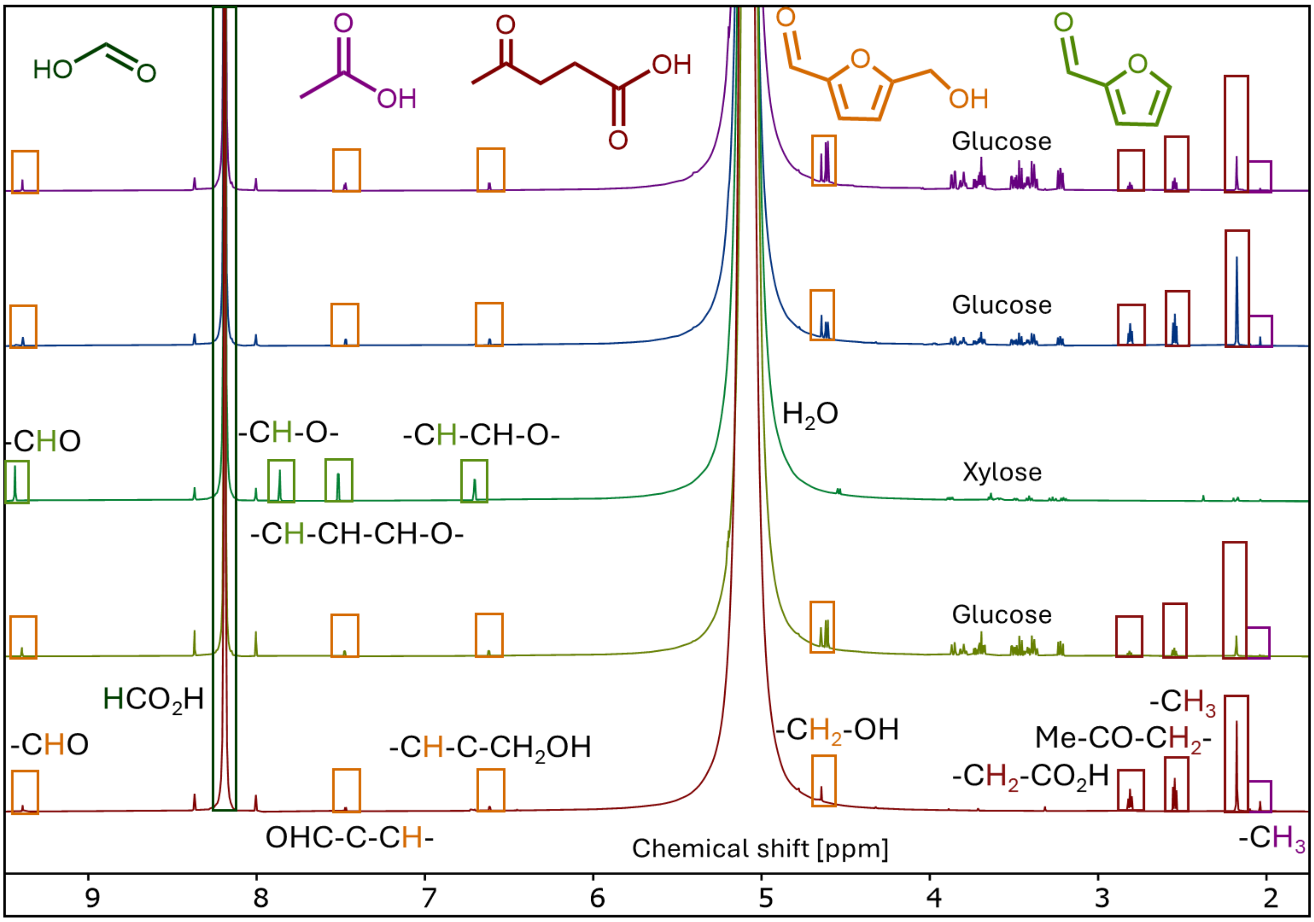

Figure S8: ^{1}H-NMR (D_2O) spectra of sugar variation reactions (from bottom to top: fructose, glucose, xylose, sucrose, cellobiose) (0.1 mol L^{-1} sugar, H_2O, pH=1 (FA), 180°C, 1 hour, p = 40 bar N_2).

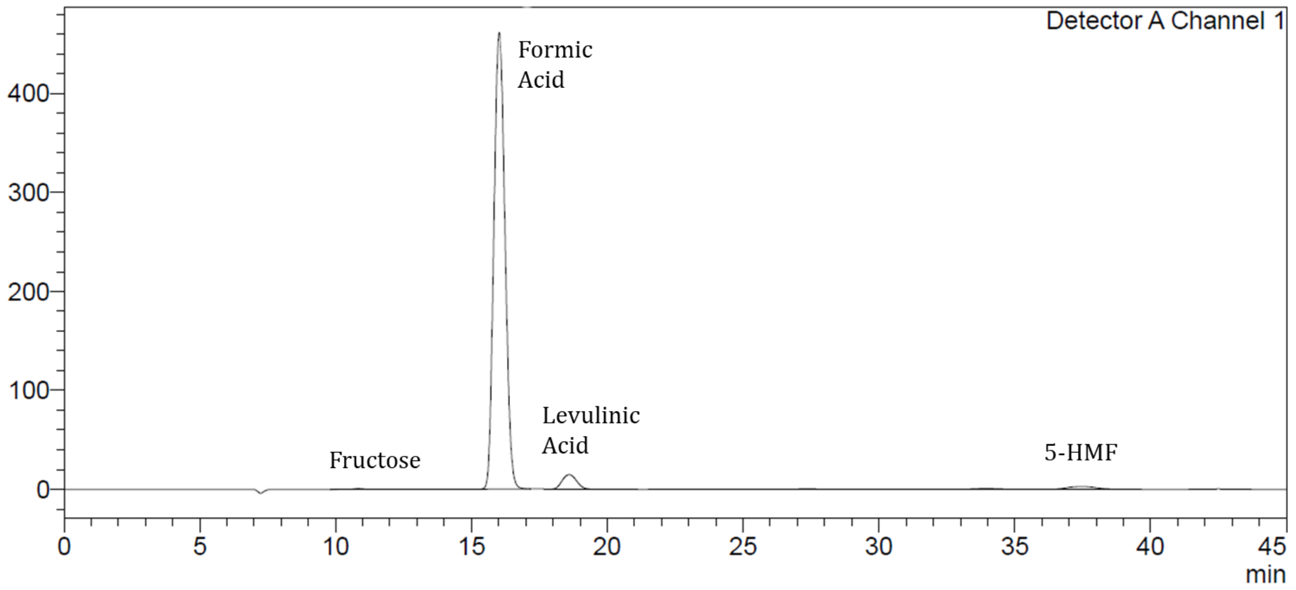

Figure S9: HPLC chromatogram of sugar variation reaction using fructose (0.1 mol L^{-1} fructose, H_2O, pH=1 (FA), 180°C, 1 hour, p = 40 bar N_2)

Table S4: Product yields of LA syntheses from fructose in the 600 mL autoclave for different reaction times (0.1 mol $^{-1}$ fructose, pH = 1 (FA); T= 180°C, p = 30 bar N_2)

**Reaction time [h]**	**LA yield [mol%]**	**Humin yield [wt%]**
2	31.0%	5.2%
3	41.0%	10.1%
4	42.0%	11.5%
5	40.0%	11.8%

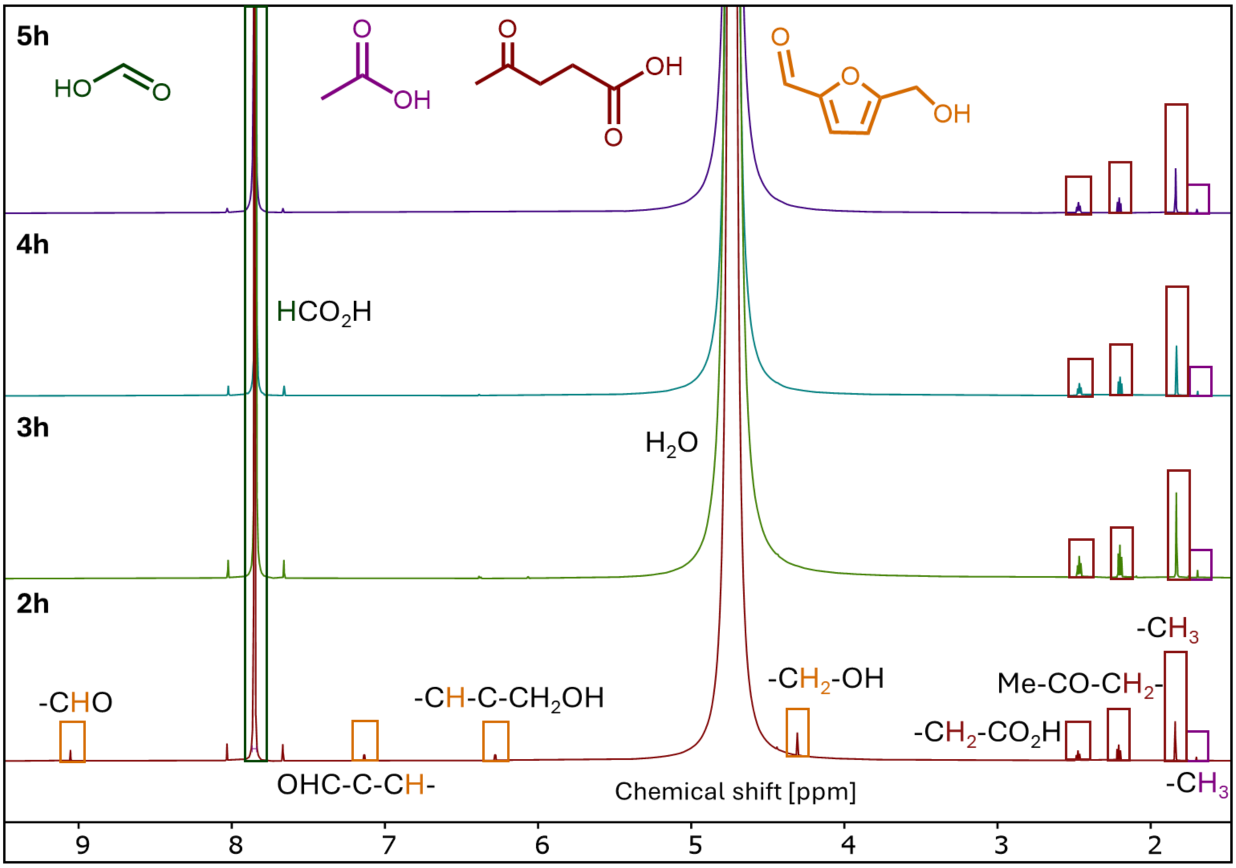

Figure S10: ^{1}H-NMR (D_2O) spectra of fructose conversions in the 600 ml autoclave with different reaction times (from bottom to top: 2,3,4 and 5 hours) (0.1 mol L^{-1} fructose, pH = 1 (FA); T= 180°C, p = 30 bar N_2)

Table S5: Elemental analyses of humins synthesized in the 600 ml autoclave with different reaction times (0.1 mol L^{-1} fructose, pH = 1 (FA); T= 180°C, p = 30 bar N_2).

**Elemental Analysis**	2h	3h	4h	5h
**C (w%)**	59.97	61.16	63.12	63.24
**H (w%)**	3.88	3.93	4.1	4.19
**O (w%)**	34.27	32.97	31.18	30.57
**Sum (w%)**	98.11	98.05	98.39	97.99

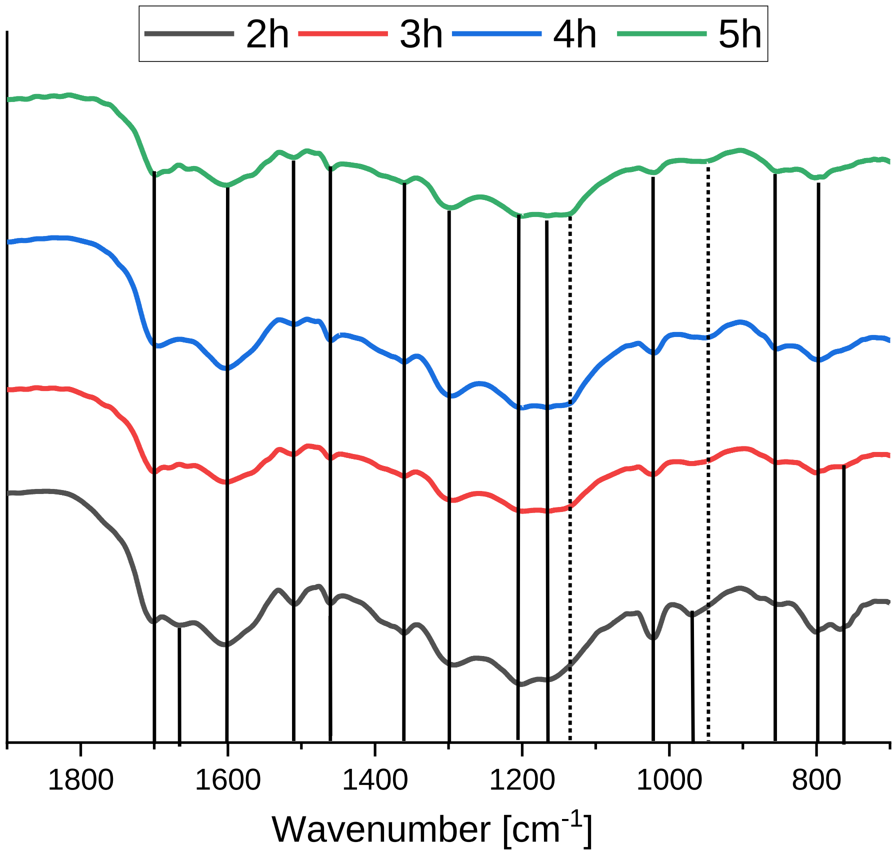

Figure S11: IR spectra of humins synthesized in the 600 ml autoclave with different reaction times (0.1 mol L^{-1} fructose, pH = 1 (FA); T= 180°C, p = 30 bar N_2).

Table S6: Vibrational band assignment of humins in included IR spectra

Wavenumber (cm^{-1})	Assignment
**765+800**	C-H Out of plane vibration substituted furan ring
**855+880**	C-H Tri-substituted alkenes
**950+970**	C-H vibration furan ring
**1025**	C-O stretch vibration
**1085**	C-O-C ether vibration
**1135+1160+1200**	C-O-C deformation vibration furan ring
**1300**	C-H rocking vibration
**1360**	C-C framework vibration (furan) C6 sugars
**1395**	C-C framework vibration (furan) C5 sugars
**1460**	C-H aliphatic chain vibration
**1510**	C=C vibration aromatic double bonds of poly substituted furans
**1600**	C=C stretch vibration conjugated with carbonyl
**1670**	C=O carbyonyl, aldehyde vibrations
**1700**	C=O stretch of acids, aldehydes and ketons

5. Cyclic Levulinic acid production (LPC)

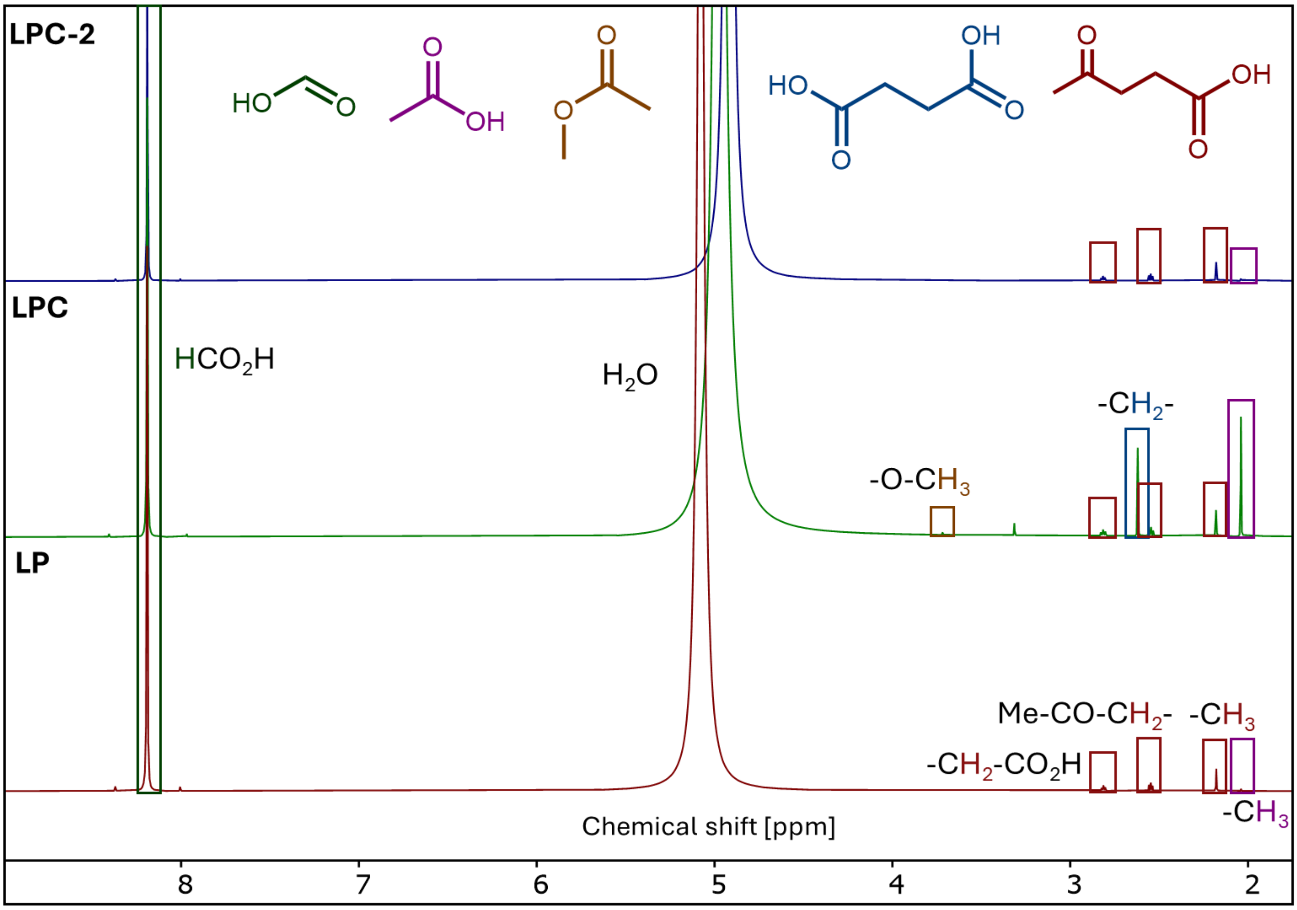

*Figure S12: ^{1}H-NMR (D_2O) comparison of **LP**, **LPC** and **LPC-2** (180°C, 0.1 mol L^{-1} fructose, pH = 1 FA, t = 4 h, p=30 bar N_2).*

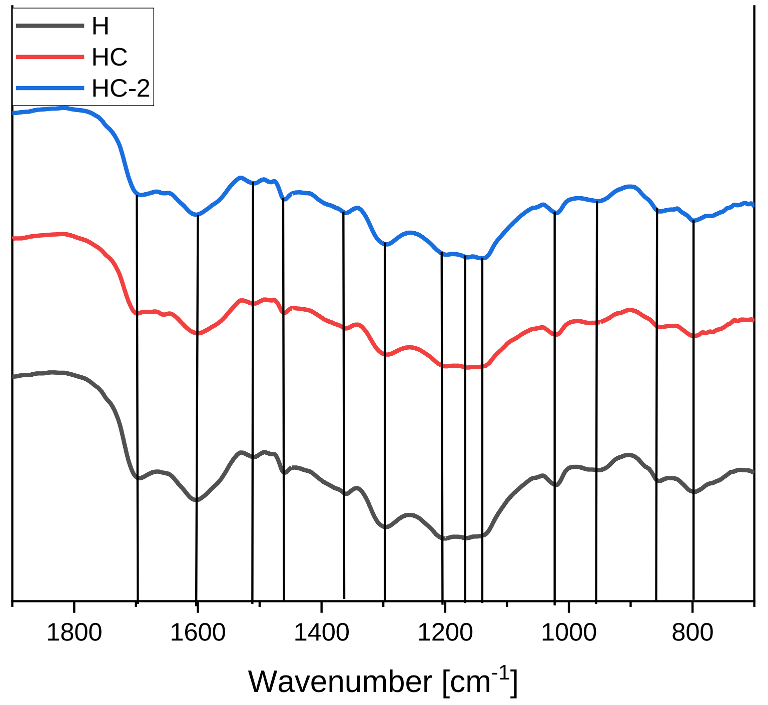

*Figure S13: Comparison of the IR spectra of the humins **H**, **HC** and **HC-2** (180°C, 0.1 mol L^{-1} fructose, pH = 1 FA, t = 4h, p=30 bar N_2).*

6. Characterization of HPA-2

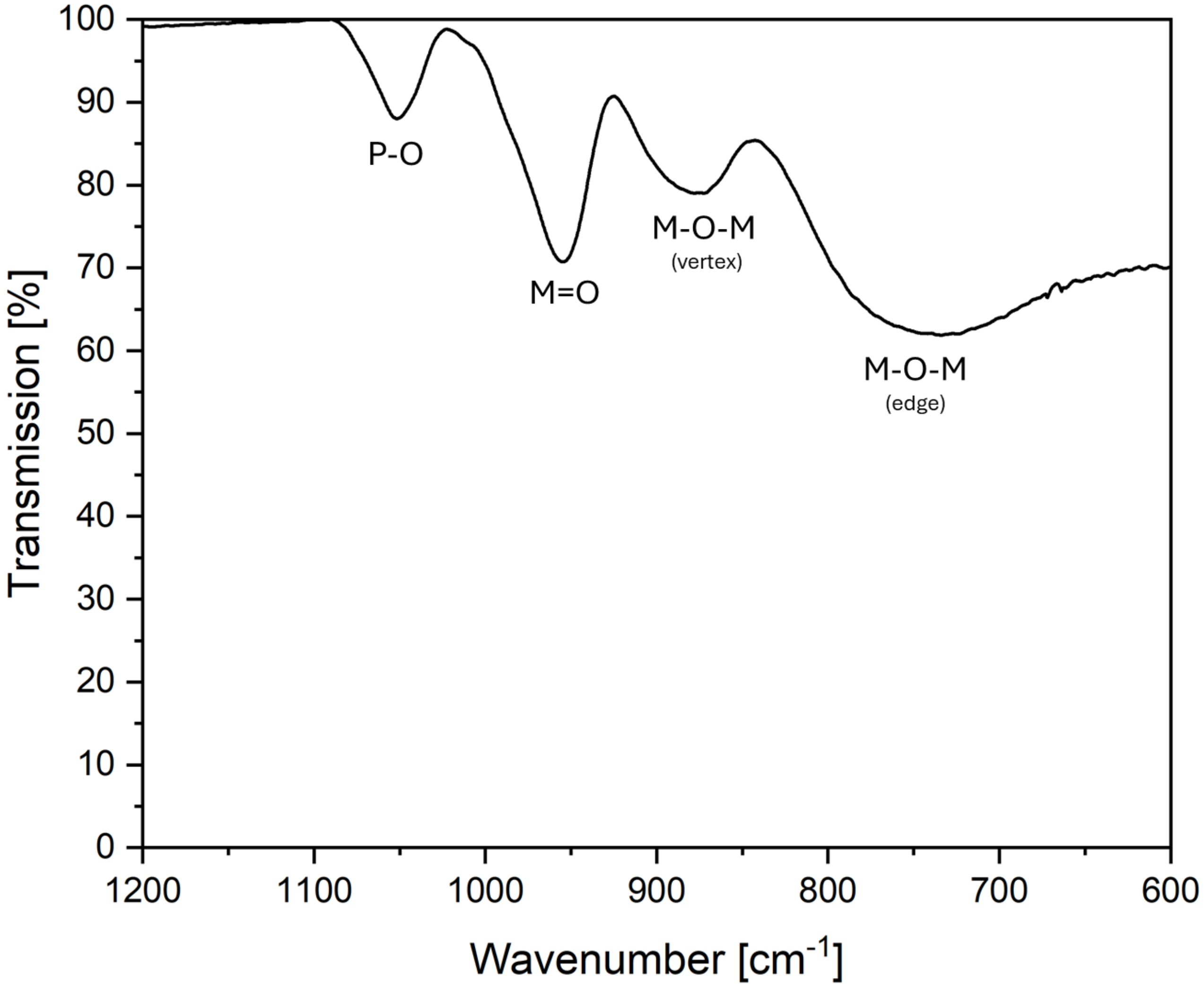

Figure S14: IR-spectrum of the fresh HPA-2 catalyst.

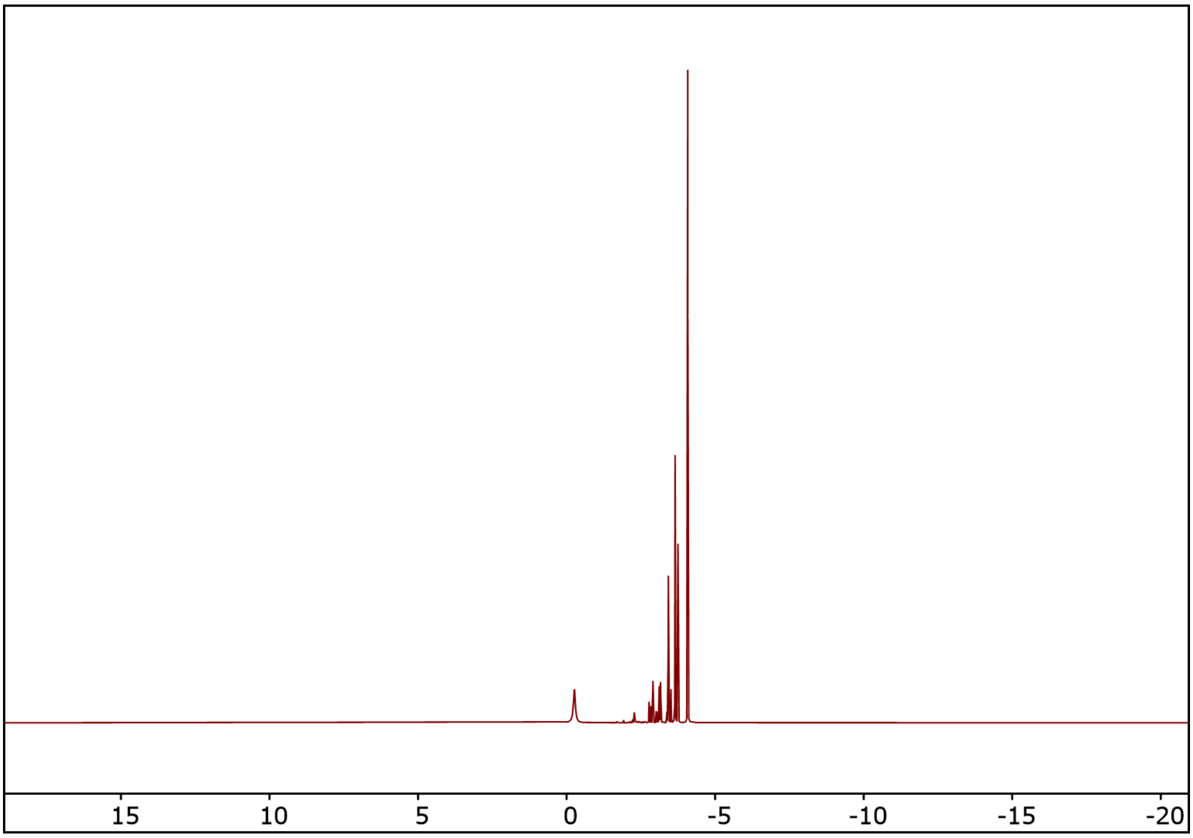

Figure S15: ^{31}P-NMR (Acetone-d_6) of the fresh HPA-2 catalyst.

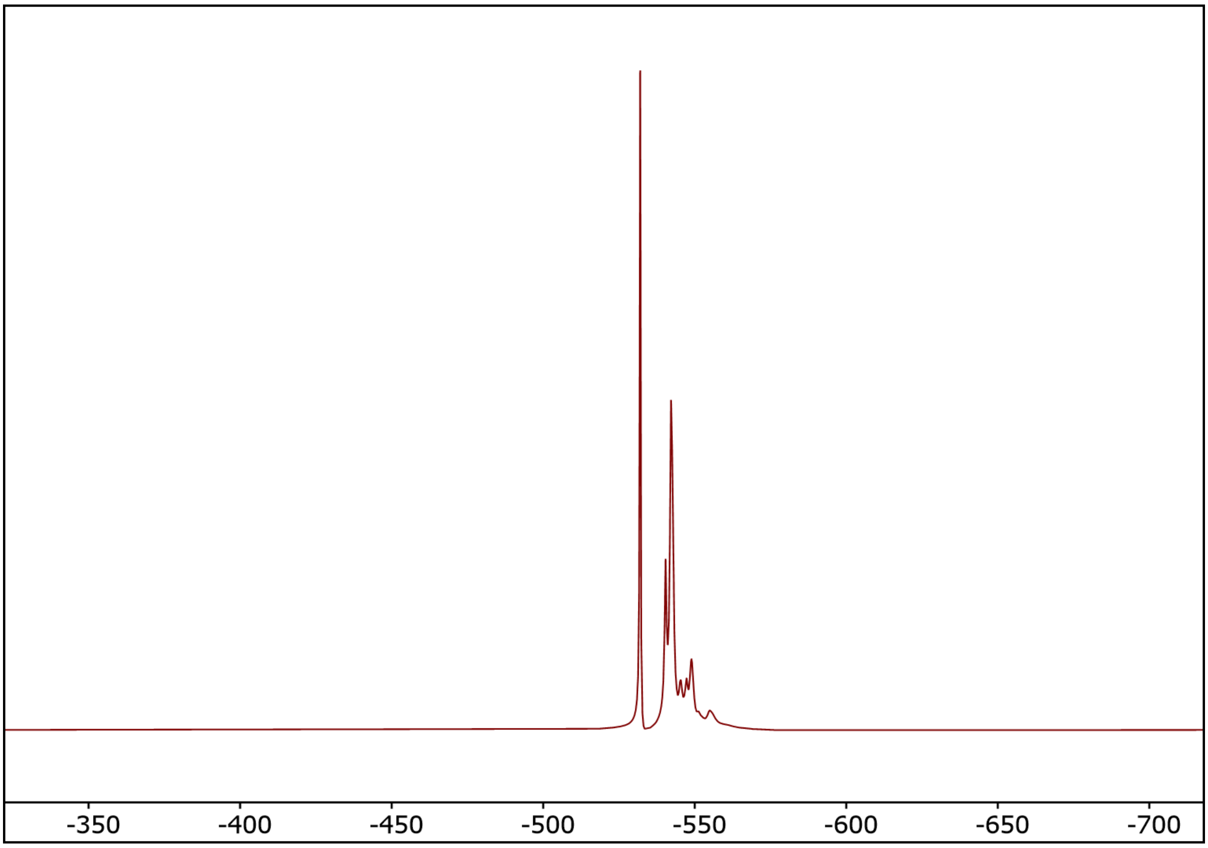

Figure S16: ^{51}V-NMR (Acetone-d_6) of the fresh HPA-2 catalyst.

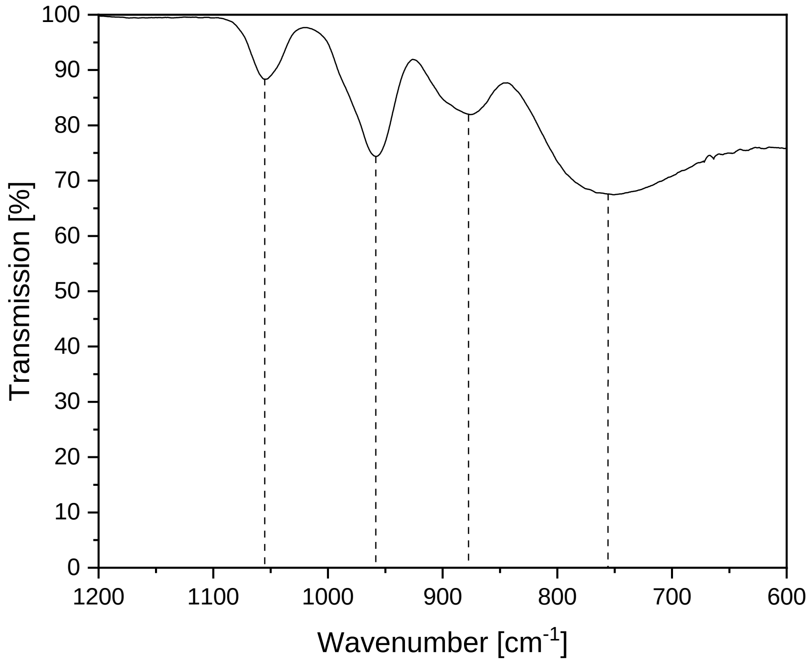

Figure S17: *FT-IR (ATR) spectrum of the used HPA-2-catalyst. Vibration modes: 1055 cm^{-1} (P-O), 958 cm^{-1} (M=O$_t$), 877 cm^{-1} ((M-O-M)$_{vertex}$), 756 cm^{-1} ((M-O-M)$_{edge}$).*[1]

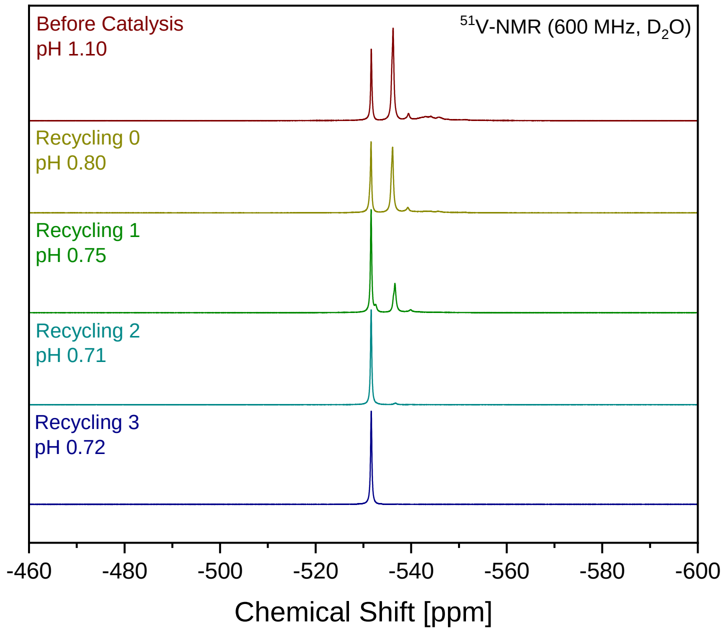

Figure S18**:** *^{51}V-NMR spectra from recycling experiments for SCO of glucose-based humin using HPA-2.*

7. Equations

Sugar conversions x_s were determined through equation S1:

$$x_s = \frac{c_{s,0} - c_s}{c_{s,0}} * 100 \tag{S1}$$

With $c_{s,0}$ being the concentration before and c_s the concentration after reaction.

The yields Pi of the products of humin conversion CH $Y_{CH,Px}$ were determined with equation S2:

$$Y_{CH,Px} = \frac{m_{Px}}{m_{Px,max}} \tag{S2}$$

With m_{Px} being the mass of the products and $m_{Px,max}$ being the maximum possible mass.

m_{Px} was determined through the multiplication of the measured density of the reaction solution with the concentration and molar mass of the reaction product. This accounted for deviations in the density through the different densities of the reaction products.

For the calculation of $m_{Px,max}$ equation S3 was used:

$$m_{Px,max} = \frac{n_{H,C}/N_{C,Px} * M_H}{m_H + m_{HPA-2} + m_{H_2O}} \tag{S3}$$

$N_{C,Px}$ here is equivalent to the number of carbons contained in the reaction product and $n_{H,C}$ is the amount of carbon contained in humin H, which was determined using equation S4:

$$n_{H,C} = \frac{C_H * m_H}{M_C} \tag{S4}$$

With C_H being the mass percentage of carbon determined by elemental analysis m_H the mass of humin H and M_C being the mass of a carbon atom.

References

[1] A. Wassenberg, T. Esser, M.J. Poller, D. Voß, J. Albert, Humin-free synthesis of levulinic acid from fructose by using heteropolyacid catalysts, Biofuels, Bioproducts and Biorefining 18 (2024). https://doi.org/10.1002/bbb.2654.
